# Knowledge and social relatedness shape research portfolio diversification

**DOI:** 10.1038/s41598-020-71009-7

**Published:** 2020-08-28

**Authors:** Giorgio Tripodi, Francesca Chiaromonte, Fabrizio Lillo

**Affiliations:** 1 Scuola Normale Superiore, 56126 Pisa, Italy; 2 Institute of Economics and EMbeDS, Scuola Superiore Sant’Anna, 56127 Pisa, Italy; 3 Department of Statistics, The Pennsylvania State University, University Park, PA 16802 USA; 4 Department of Mathematics, University of Bologna, 40126 Bologna, Italy

**Keywords:** Scientific data, Statistics, Complex networks

## Abstract

Scientific discovery is shaped by scientists’ choices and thus by their career patterns. The increasing knowledge required to work at the frontier of science makes it harder for an individual to embark on unexplored paths. Yet collaborations can reduce learning costs—albeit at the expense of increased coordination costs. In this article, we use data on the publication histories of a very large sample of physicists to measure the effects of knowledge and social relatedness on their diversification strategies. Using bipartite networks, we compute a measure of topic similarity and a measure of social proximity. We find that scientists’ strategies are not random, and that they are significantly affected by both. Knowledge relatedness across topics explains $\approx 10\%$ of logistic regression deviances and social relatedness as much as $\approx 30\%$, suggesting that science is an eminently social enterprise: when scientists move out of their core specialization, they do so through collaborations. Interestingly, we also find a significant negative interaction between knowledge and social relatedness, suggesting that the farther scientists move from their specialization, the more they rely on collaborations. Our results provide a starting point for broader quantitative analyses of scientific diversification strategies, which could also be extended to the domain of technological innovation—offering insights from a comparative and policy perspective.

## Introduction

The activities of scientists and innovators often span several areas, with choices of research endeavours driven by a variety of factors. The “essential tension” between exploration and exploitation described by Kuhn certainly characterizes research careers^1^, but scientists can evolve ways to handle this trade-off. On the one hand, advances in science and technology create a “burden of knowledge”^2^; the sheer amount of information required to move forward has grown, and larger educational costs may force scientists and innovators towards a narrower specialization. On the other hand, contemporary science is dominated by teams that bring together different expertise—albeit at a cost in terms of coordination and credit sharing^3^. This article focuses on the analysis of scientists’ research portfolio, investigating the roles of knowledge relatedness (among research topics) and social relatedness (among authors), as well as their interaction, as drivers of diversification.

Recent efforts to better characterize patterns in research and innovation activities produced valuable insights. For instance, based on a knowledge network created using MEDLINE articles annotated with chemical entities, Foster et al.^4^ quantitatively analyzed the dichotomy between exploration and exploitation. According to their taxonomy, each new article can expand or consolidate the knowledge space by generating a new chemical relationship (i.e., a new combination) or contribute to an existing one. Results show that research strategies (i.e., the types of articles produced) are stable over time and exploitation is preferred over exploration, despite a growing number of opportunities. Exploration is riskier, with rewards (i.e., citations) that are higher but insufficient to compensate the risk. In the domain of physics, Pan et al.^5^ focused on the temporal evolution of interdisciplinary research. The authors constructed and analyzed yearly snapshots of the connections among physics sub-fields uniquely identified through PACS codes. Results show that connectivity, and thus interdisciplinarity within physics, increased—but in a non-random way that reflects the hierarchical structure of sub-fields. In particular, *condensed matter* and *general physics* acted as hubs for the increasing number of connections. Recently, Sun et al.^6^ proposed a novel framework, based on time-varying networks, to track knowledge flows within and across physics sub-fields. Such a method is able to highlight the increasing general trend towards interdisciplinary research as well as identify interesting patterns of influence among sub-fields over time. More directly related to our purposes, recent works^7–9^ collected compelling empirical evidence on physicists’ research endeavours. Battiston et al.^7^ provided a comprehensive census of academic physicists active in recent decades. The authors charted a thorough picture of the evolution of various fields in terms of number of scientists, productivity (including impact and recognitions such as Nobel prizes), team size and role of chaperones—highlighting a rich heterogeneity among specializations. Moreover, Battiston et al.^7^ mapped “migration” flows by comparing the field in which a given scientist published her first paper with the one characterizing her later research interests.

Also Aleta et al.^8^ mapped flows among physics sub-fields, with the aim of investigating the “essential tension” in the evolution of scholars’ research interests. The authors defined a measure of exploration comparing early- and late-career ranges of actives, and tracked flows using origin-destination matrices among fields. Results suggest a preference for exploration over exploitation, but concentrated within the same broad area of research, and non-random transitions among different areas. Jia et al.^9^ observed that the frequency of scientists decays exponentially as one considers increasing degrees of change in interests. In order to reconstruct the macroscopic patterns that drive such evolution, the authors proposed a random walk model over a stylized knowledge space, which reproduces empirical observations thanks to the inclusion of key features such as heterogeneity, subject proximity and recency. Finally, Zeng et al.^10^ analysed the dynamics of “topic switching” by exploring co-citation networks. Results suggest a growing propensity to switch among topics but also that such a strategy might hamper productivity, especially for early-career researchers.

Despite the growing body of evidence and stylized facts provided by this literature, much remains to be done to disentangle and quantify the roles of different contributing factors. To make progress in this direction, we investigate scientists’ research portfolio diversification by quantifying potential drivers of exploration, or, to put it differently, the hurdles faced by scientists when they move out of their immediate specialization. We use a network approach to compute a measure of similarity among research sub-fields, define a measure of social relatedness and track of scientists’ diversification patterns. We build our empirical strategy upon the intuition of Breschi et al.^11^, who used patent data to explore the nature and degree of coherence in firms’ technological diversification.

Our analysis proceeds as follows. First, we test and reject the hypothesis that research portfolio diversification is random. Second, we use regression techniques to characterize how subject and social proximity affect diversification, controlling for possible confounding factors. Third, we quantify the relative importance of our relatedness measures. We provide robust empirical evidence that knowledge and social relatedness are both significant statistical predictors of diversification, as is their interaction – which corroborates the notion that collaborations modulate knowledge acquisition, especially when scientists move far from their own specialization. Like many of the articles mentioned above, we analyze data concerning physicists. This focus is due in part to the central role of physics among the *hard* sciences, and in part to the reliability of data collected labeling articles through the PACS codes. Nevertheless, our approach is fully general and could be used in different domains.

## Results

### Data description

We use the American Physical Society (APS) dataset to reconstruct the activities of 197,682 physicists who published at least one paper in one of the APS outlets in the period ranging from 1977 to 2009 (see the “Data” section for details). All articles in APS journals are classified according to hierarchical codes that map into physics fields and sub-fields (i.e., PACS codes). For our analyses (see the “Modeling and assessment of predictors’ contributions” section), we filter out authors and sub-fields that appear only sporadically in the data. Specifically, we focus on 105,558 authors who published at least two articles, covering a minimum of two sub-fields over a restricted set of 68 PACS which appear in at least four articles.

**Figure 1:**
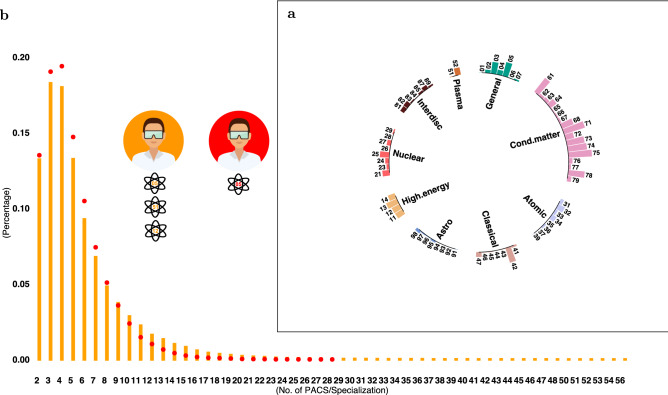
Popularity of fields and scientists’ degree of diversification/specialization. (**a**) Circular bar-chart showing the number of articles assigned to each sub-field in the one-digit PACS codes, taking into account their hierarchical structure. The chart highlights the popularity of *Condensed Matter* research in both size and scope. (**b**) Distribution of scientists’ degree of diversification (the number of sub-field they explored; orange bars) and of their relative specialization (the number of sub-fields in which they have a scientific advantage; red dots). Scientists explore several sub-fields, but specialize in only a few—despite the existence of some individuals with a truly interdisciplinary path, by and large research portfolios are fairly limited in scope. Inset: pictorial description of a scientist who explored three sub-fields (orange) but has only one specialization (PACS 05: red).

Figure 1 provides a general description of the data and some insights. Figure 1**a** shows the popularity, in terms of number of articles, of fields and sub-fields (one- and two-digit level PACS codes, respectively). As expected, PACS popularity is highly heterogeneous and reflects the prominence of *condensed matter* research in the last decades. Figure 1**b** shows scientists’ degree of diversification and their relative specialization, as defined in the “Scientists’ specializations” section. The research portfolio of most scholars in our dataset is fairly limited in scope, with a large majority of scientists diversifying in no more than 5 sub-fields. The choice of subjects, however, is not random—as we demonstrate in the next section.

### Diversification is not random

Do scientists, much like firms^11,12^, shape their research portfolios based on specific strategies and constraints? To address this question quantitatively, we draw a parallel with ecology: as species may co-occur in distinct sites, sub-fields may overlap in research portfolios. Measuring the relatedness of species based on their geographical co-occurrence is analogous to measuring the relatedness of sub-fields based on their overlap in scientists’ ranges of activity. Thus, the PACS-Authors binary bipartite network resembles a presence-absence matrix^13^. The monopartite projection of this bipartite network (see the “Monopartite projections of bipartite networks” section) on the PACS layer carries a critical piece of information: for each pair of PACS, it tells us how many scientists are active in both sub-fields irrespective of the number of articles, drawing a diversification network.

We can assess this network contrasting it against an appropriate null model. Which sub-fields overlaps are over- or under-represented relative to what we would expect under the assumption that scientists picked research topics at random, but taking into account the popularity of sub-fields? Under a random model, the probability that *x* scientists are active both in sub-field *a* and in sub-field *b*, given that $S_a$ and $S_b$ scientists are active in these sub-fields, follows a hypergeometric distribution^14^

1 $$\begin{aligned} P(X=x) = \frac{\left( {\begin{array}{c}S_a\\ x\end{array}}\right) \left( {\begin{array}{c}S-S_a\\ S_b-x\end{array}}\right) }{\left( {\begin{array}{c}S\\ S_b\end{array}}\right) } \end{aligned}$$

where *S* is the total number of scientists in the sample.

**Figure 2:**
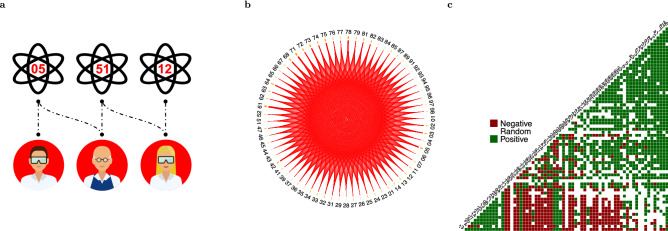
Diversification patterns. (**a**) A stylized picture of the original PACS-Authors bipartite network representing scientists’ diversification patterns. (**b**) The diversification network (the monopartite projection on PACS): links represents the number of scientists active in each pair of sub-fields. (**c**) Visual summary of the hypergeometric test, providing evidence of the coherent nature of scientists’ diversification choices: 72% of pairs are classified as non-random ($p<0.05$ after Bonferroni correction).

Figure 2 describes the steps of our procedure. Starting from the bipartite network (panel **a**), we derive its monopartite projection (panel **b**) and test whether the resulting structure is non-random, summarizing statistically validated diversification patterns (panel **c**). Out of 2,278 pairs of PACS, 72% are classified as non-random with a Bonferroni-corrected *p*-value $<0.05$. Of these, 1,151 pairs show a positive association and 486 a negative one. Given the severity of the Bonferroni correction (i.e., power decreases significantly as the number of tests increases) and possible issues related to dependency, we also employ the *False Discovery Rate* (FDR) Benjamini-Hochberg and Benjamini-Yekutieli corrections (see section S2 and Table S2). These results strongly support a coherent nature of scientists’ diversification choices, but do not provide a direct quantification of the role played by specific features in shaping such coherence. Next, we investigate potential drivers of diversification considering measures of cognitive and social proximity.

### Knowledge and social relatedness predict diversification

**Figure 3:**
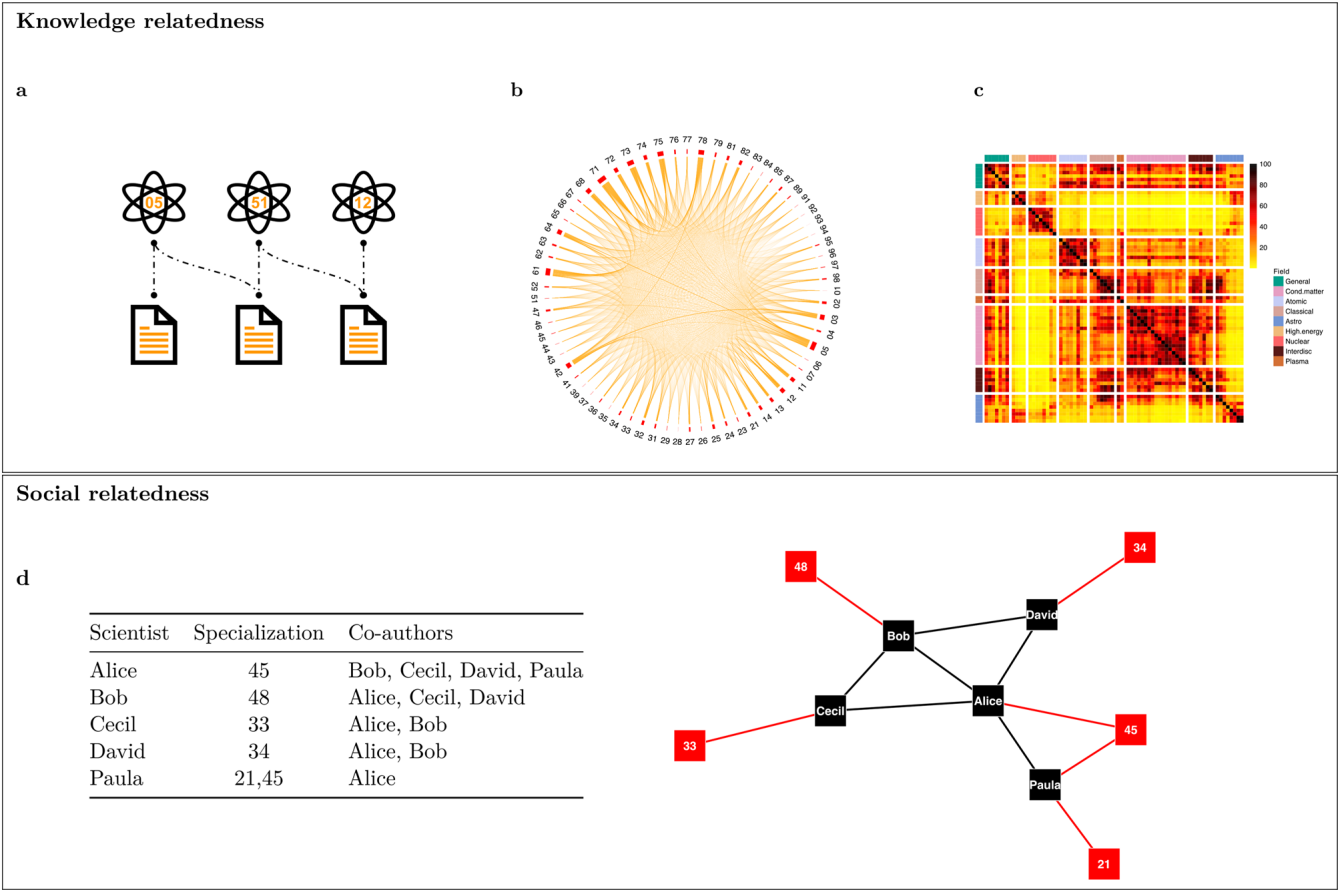
Knowledge and social relatedness. (**a**) A stylized example of the bipartite PACS-Articles network. (**b**) The PACS co-occurrence network (monopartite projection on PACS codes). (**c**) The cosine similarity matrix, which “maps” the physics knowledge space and identifies clusters corresponding to fields. (**d**) A table illustrating how co-authorship and specialization information are combined to produce the augmented co-authorship network shown in the figure, which includes nodes attributes (specializations). The nodes represent individual scientists (in black) and specializations (in red). Our measure of social relatedness ($SR_{ib}$) is defined as a dummy that captures whether scholar *i* can reach a certain sub-field *b* through social interactions; $SR_{ib} = 1$ if $d(i,b)=2$, where *d*(*i*, *b*) is the geodesic distance between scholar *i* and sub-field *b*. For instance, $SR_{David,45}=1$ since David could directly exchange knowledge with Alice (specialized in sub-field 45), while $SR_{David,21}=0$.

The relationships among scientific fields, like those among technologies, can be mapped using network science tools. To chart a knowledge space we need a measure of distance between fields. Several different metrics have been proposed to quantify the relatedness of technologies or scientific domains (see Bowen et al.^15^ for a review). When we consider the monopartite projection on the PACS layer of the bipartite PACS-Articles network, counting the co-occurrences of all pairs of PACS produces a first approximation of the relatedness of sub-fields. A similar approach was used by Lamperti et al.^16^ for patent data. However, we need a measure of proximity that: (i) does not depend on the absolute popularity of the fields, and (ii) is symmetric. The most straightforward metric that fulfils both requirements is the cosine similarity (see Fig. 3**a**−**c**, “Measures of knowledge and social relatedness” section). As expected, the proximity matrix has a clear hierarchical block structure, with blocks largely overlapping with fields. Interestingly, several off block elements show the proximity of sub-fields belonging to different PACS fields.

As science becomes an increasingly “social” enterprise, it is also important to capture the relatedness of scholars, which can be done by analysing co-authorships^3^. Similar to what we did for knowledge relatedness, we construct a measure of social relatedness starting from the bipartite Authors-Articles network. The monopartite projection on the Authors layer defines the co-authorship network from which we compute our desired metric. In addition, to investigate whether diversification is associated with the exploitation of social relationships, we include information on authors’ specialization as node attributes in the network and we introduce a dummy $SR_{ib}$ equal to 1 if scientist *i* can reach sub-field *b* through direct social interactions (see Fig. 3**d**, the “Measures of knowledge and social relatedness” section).

Next, we evaluate the effects of knowledge and social relatedness on diversification with logistic regressions. The binary dependent variable encodes whether a scientist is active in a sub-field, the main explanatory variables are our measures of cognitive and social proximity, and a control is introduced for the core field. In practice, each scientist is assigned to a core sub-field (specialization) and can possibly diversify in one or more target sub-fields different from her own (see the “Scientists’ specializations” section). In this first set of regressions, each scientist appears 67 times, one for every possible target PACS different from her own specialization (see the “Modeling and assessment of predictors’ contributions” section for more details).

**Figure 4:**
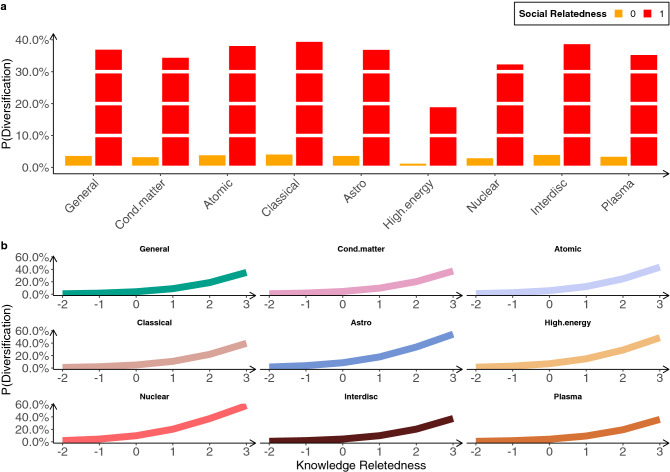
Probabilities of scientists diversifying in a sub-field different from their own specialization. Predicted probabilities of a scientist being active in a sub-field different from her own specialization as a function of (**a**) (binary) social relatedness, and (**b**) (standardized) knowledge relatedness. Results are obtained by fitting a logistic regression with only one control variable—the scientist’ core field. All coefficients are statistically significant ($p<0.01$).

Figure 4 provides evidence that both social and knowledge relatedness are associated with scientists’ diversification strategies. Social relatedness matters irrespective of the field, as scientists who can acquire new knowledge through social relationships are more likely to be active in a sub-field different form their own specialization (panel **a**). Also knowledge relatedness increases the probability of a scientist being active out of her own specialization, and again this is true for all fields (panel **b**). These results strongly suggest that cognitive and social proximity do contribute to shaping diversification strategies.

### Model extensions and robustness checks

To move further in our investigation of research portfolio diversification, we broaden our analysis in several ways. *First*, we expand our logistic regression model including a larger set of control variables, such as the number of co-authors or the popularity and citations of the target sub-field (see Table S3 for a complete list). All numerical variables in the expanded model are normalized, and log-transformed to reduce right-skew when necessary (see the “Modeling and assessment of predictors’ contributions” section for more details). Since the effect of knowledge relatedness on the probability of diversification may be modulated by social relatedness, we also include an interaction term in our analysis.

*Second*, we tackle two potential limitations of our original analysis; that is, defining a single specialization for each scientist (while core specializations may actually be multiple), and not separating sub-field movements within and between fields, i.e., one-digit PACS codes (which may be differently affected by various features). We run additional model fits allowing scientists to have multiple specializations (see the “Scientists’ specializations” section) and separating within and between field diversification. Specifically, we perform the following fits: (i) single specialization with full diversification, (ii) multiple-specialization with full diversification, (iii) single specialization with within field diversification, (iv) multiple specialization with within field diversification, (v) single specialization with between field diversification and (vi) multiple specialization with between field diversification.

*Third*, we account for the fact that the data employed in our fits are “clustered”, with several observations associated to each scientist and a potential heteroskedasticity across clusters/scientists. We estimate clustering-robust standard errors using the clustered sandwich estimator from the **R** package *sandwich*^17^.

**Figure 5:**
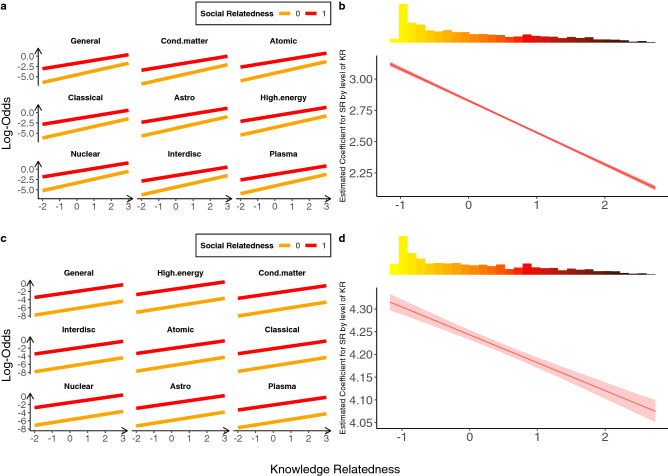
Scientists’ research portfolio diversification: full diversification, single and multiple specialization. (**a**) Log-odds as function of (binary) social relatedness and (standardized) knowledge relatedness, accounting for multiple control variables, for the single specialization specification (i). (**b**) Estimated coefficient for social relatedness conditional on knowledge relatedness, and distribution of knowledge relatedness (on top, similarity color coded as in Fig. 3**c**, for the single specialization specification (i)). (**c**,**d**) Same as (**a**) and (**b**) for the multiple specialization specification (ii).

Fits for specifications (i)–(iv), all including the interaction between knowledge and social relatedness and clustering corrected standard errors, are summarized in Table 1, confirming the high significance of the relatedness metrics in shaping research diversification. Figure 5 focuses on the full diversification case. Panels **a** (single specialization, (i)) and **c** (multiple specialization, (ii)) show the log-odds difference in the probability of diversification as a function of knowledge and social relatedness, accounting for all controls. Social relatedness positively affects the chances of diversification and the effect is moderated by knowledge relatedness in both specifications, though more markedly in (i) than in (ii). Panels **b** (for (i)) and **d** (for (ii)) further illustrate this, showing how the estimated coefficient of social relatedness decreases as knowledge relatedness increases. This result indicates that when diversifying toward “close” sub-field, the role of social relatedness becomes less crucial.

**Table 1:** Regression results.

	Dependent variable: prob (diversification)					
	Full diversification		Within field diversfication		Between field diversfication	
	Single	Multiple	Single	Multiple	Single	Multiple
	(i)	(ii)	(iii)	(iv)	(v)	(vi)
Knowledge relatedness	0.936***	0.688***	0.184***	0.121***	0.702***	0.511***
	(0.003)	(0.009)	(0.005)	(0.013)	(0.003)	(0.011)
Social relatedness	2.827***	4.243***	2.272***	3.968***	2.914***	4.284***
	(0.006)	(0.019)	(0.008)	(0.021)	(0.008)	(0.021)
Field core-atomic	$-$0.332***	$-$0.428***	0.056**	$-$0.276***	$-$0.303***	$-$0.385***
	(0.010)	(0.007)	(0.025)	(0.021)	(0.010)	(0.008)
Field core-classical	$-$0.490***	$-$0.477***	$-$1.001***	$-$0.932***	$-$0.313***	$-$0.328***
	(0.010)	(0.007)	(0.029)	(0.023)	(0.010)	(0.008)
Field core-cond. matter	$-$1.088***	$-$0.761***	$-$1.110***	$-$0.892***	$-$1.263***	$-$0.903***
	(0.012)	(0.009)	(0.024)	(0.020)	(0.017)	(0.013)
Field core-general	$-$0.722***	$-$0.537***	$-$0.927***	$-$0.823***	$-$0.632***	$-$0.422***
	(0.011)	(0.007)	(0.028)	(0.021)	(0.012)	(0.008)
Field core-hgh.energy	0.219***	0.168***	1.806***	1.176***	$-$0.360***	$-$0.060***
	(0.010)	(0.006)	(0.027)	(0.023)	(0.013)	(0.008)
Field core-interdisc	$-$0.557***	$-$0.553***	$-$0.357***	$-$0.724***	$-$0.365***	$-$0.367***
	(0.010)	(0.007)	(0.026)	(0.021)	(0.011)	(0.008)
Field core-nuclear	0.463***	0.164***	0.969***	0.692***	0.068***	$-$0.161***
	(0.010)	(0.006)	(0.024)	(0.021)	(0.011)	(0.009)
Field core-plasma	$-$0.269***	$-$0.419***	$-$0.155**	$-$0.361***	$-$0.074***	$-$0.256***
	(0.013)	(0.008)	(0.068)	(0.058)	(0.015)	(0.009)
# of PACS	0.882***	0.806***	0.769***	0.497***	1.003***	0.944***
	(0.002)	(0.003)	(0.005)	(0.004)	(0.004)	(0.004)
# of papers	0.010***	0.113***	0.065***	0.252***	$-$0.032***	0.050***
	(0.002)	(0.003)	(0.005)	(0.004)	(0.004)	(0.004)
# of co-authors	$-$0.406***	$-$0.347***	$-$0.240***	$-$0.145***	$-$0.488***	$-$0.444***
	(0.002)	(0.004)	(0.004)	(0.004)	(0.003)	(0.006)
PACS target popularity	1.130***	0.611***	1.370***	0.774***	1.108***	0.559***
	(0.002)	(0.002)	(0.005)	(0.003)	(0.003)	(0.002)
$\Delta$ crowd	0.239***	0.358***	0.131***	0.345***	0.320***	0.393***
	(0.002)	(0.003)	(0.003)	(0.003)	(0.003)	(0.003)
$\Delta$ PACS citations	$-$0.273***	$-$0.332***	$-$0.208***	$-$0.313***	$-$0.369***	$-$0.354***
	(0.002)	(0.003)	(0.004)	(0.003)	(0.004)	(0.003)
$\Delta$ field citations	$-$0.156***	$-$0.070***	✗	✗	$-$0.196***	$-$0.143***
	(0.004)	(0.004)	(✗)	(✗)	(0.006)	(0.005)
KR:SR	$-$0.255***	$-$0.061***	$-$0.047***	$-$0.001	$-$0.234***	$-$0.067***
	(0.004)	(0.010)	(0.007)	(0.013)	(0.005)	(0.011)
Constant	$-$3.812***	$-$5.903***	$-$1.882***	$-$4.250***	$-$4.168***	$-$6.165***
	(0.010)	(0.020)	(0.022)	(0.028)	(0.010)	(0.022)
Observations	7,072,386	35,968,615	1,000,230	5,407,404	6,072,156	30,154,990
Log kikelihood	$-$1,086,281.000	$-$7,303,198.000	$-$334,697.300	$-$2,166,803.000	$-$716,398.900	$-$4,971,497.000
Akaike inf. crit.	2,172,600.000	14,606,434.000	669,430.600	4,333,642.000	1,432,836.000	9,943,033.000

Coefficients of the logistic regressions of Eq. (8), i.e. the model including the interaction term between knowledge and social relatedness, under different specialization settings. The table reports clustering corrected standard errors (in parenthesis) and significance level: *p < 0.1; **p < 0.05; ***p < 0.01. ✗: variable not included in the model.

Next, we contrast scientists moving within their specialization field (between two sub-fields, i.e. two-digit PACS codes, belonging to the same field, i.e. one-digit PACS code; e.g. PACS 12 *Specific theories and interaction models; particle systematics* and PACS 13 *Specific reactions and phenomenology*, both belonging to PACS 1 *High Energy* physics) and scientists moving out of their field and towards a completely different subject (i.e. a different one-digit PACS code). These choices may be driven by different factors. Scientists moving within their field may be less dependent on external collaborations, since such a diversification strategy requires a smaller learning effort. Our estimates do highlight differences. Looking at the within field diversification case, single specialization (Table 1, (iii)), we see that knowledge and social relatedness, as well as their interaction, are still significant—but the magnitude of the coefficients is smaller with respect to the full diversification case. When we consider multiple specialization (Table 1, (iv)), coefficients shrink even further and the interaction is no longer significant (see also Figure S2). On the contrary, looking at the between field diversification case, the general trends outlined for the full diversification case are confirmed—including the negative interaction term remaining sizeable and significant for both single and multiple specialization (see Table 1, (v) and (vi), and Figure S3).These results are in line with expectations: while having a co-author in a different sub-field may well be useful, knowledge is not a barrier to entry when scientists move within the same general area of inquiry. This explains why the interaction between social and knowledge relatedness becomes less prominent or non-significant in our estimates.

### Quantifying the relative importance of knowledge and social relatedness

Can we quantify the (relative) role of knowledge and social relatedness in explaining research portfolio diversification? How important are these quantities when evaluated in the presence of several control covariates, and under a range of model specifications? To answer these questions we follow two approaches.

**Figure 6:**
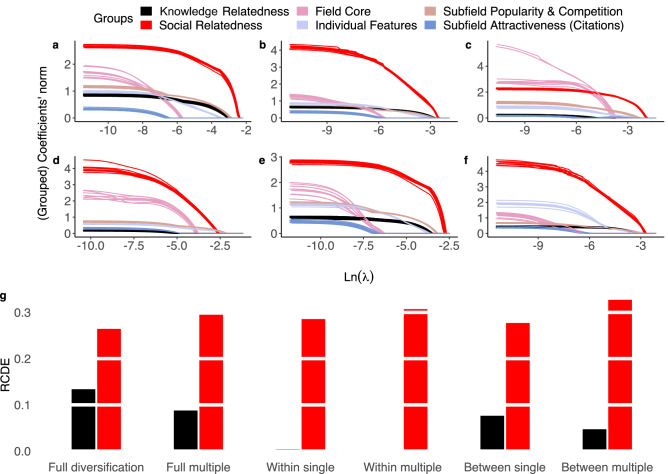
Relative importance of predictors. (**a**–**f**) Group LASSO paths for (**a**) full diversification, single specialization; (**b**) full diversification, multiple specialization; (**c**) within-field diversification, single specialization; (**d**) within-field diversification, multiple specialization; (**e**) between-field diversification, single specialization; (**f**) between-field diversification, multiple specialization. In each panel, variables in the same group are color coded, and their average coefficient norm is plotted (as a single path) against the penalty parameter (log $\lambda$). The multiple paths for each color correspond to separate group LASSO runs on 10 random sub-samples of 1,000 scientists. (**g**) Relative Contributions to Deviance Explained for knowledge relatedness (black) and social relatedness (red) across all fits.

*First*, we run a LASSO feature selection procedure to gauge the relative importance and role of different predictors by tracking how they are excluded/included in a model as one varies the regularization penalty. Since our predictors include categorical variables (i.e., groups of dummies), as well as naturally grouped variables (e.g., scientists’ individual characteristics, sub-fields’ popularity and competition, etc.) we run a *group* LASSO algorithm^18^ with features grouped as shown in Table S3. Moreover, to counteract collinearity and finite sample issues which can render the LASSO unstable^19^, we split our data forming ten random subsamples of 1,000 scientists each, and repeat the group LASSO fit on each of the subsamples for all the considered model specifications. Figure 6**a**–**f** show the (grouped) coefficient norms as a function of the penalization parameter $\lambda$. Results clearly demonstrate the crucial role played by social and knowledge relatedness. They also confirm that the role of knowledge relatedness weakens markedly in the case of within-field diversification (panels **c** and **d**).

*Second*, we compute the *Relative Contributions to Deviance Explained* (RCDEs; see the “Modeling and assessment of predictors’ contributions” section for details). This index captures what percentage of the logistic regression deviance is captured by a predictor. Figure 6**g** strongly supports a prominent role for social relatedness, with RCDEs around or above 30% across all specifications. The RCDEs of knowledge relatedness are smaller, around 5–10%, and again become negligible in the case of within-field diversification. In summary, our results provide additional evidence that both social and knowledge proximity shape scientists’ diversification strategies, but highlight social interactions as the dominant channel through which knowledge is exchanged and acquired.

### Digging deeper: multidisciplinarity and time

Next, we tackle two additional potential limitations of our original analysis, which might overestimate the probability of diversification for truly multidisciplinary scientists and suffer from reverse causality issues. To investigate diversification into truly unexplored sub-fields, we fitted the model specification (i) (see the “Model extensions and robustness checks” section) considering scientists’ specialization (see the “Scientists’ specializations” section) and limiting their diversification choices to sub-fields in which they have no revealed scientific advantage (see section S5.1). To at least partially address causality in the effects of knowledge and social relatedness on diversification, we included a temporal dimension: we split the original dataset in three time periods, re-computed our measures of relatedness in each, and used them to predict scientists’ diversification introducing time lags (see section S5.2). In both exercises, results confirmed our previous findings: social relatedness shapes scientists’ diversification strategies more than knowledge relatedness.

Finally, and again related to time, our findings may be influenced by underlying trends in the temporal evolution of PACS co-occurrence networks—and thus knowledge proximity. A detailed study of the evolution of relationships among sub-fields, which is of course of interest *per se*, is beyond the scope of the present article. Nevertheless, to gather at least some approximate sense of its potential impact, we recomputed our measure of knowledge relatedness separately for each of the different decades in the original dataset. Based on results shown in section S4, the physics knowledge space remained rather stable over the time span considered. A valuable alternative approach to take into account the temporal evolution of the physics knowledge space is provided by Chinazzi et al.^20^

## Discussion

Scientists try to balance the “tension” between exploitation and exploration, but the exploration phase is, to some extent, constrained by the “burden of knowledge”. To tackle the rising complexity of producing new knowledge, scientists adapt their diversification strategies leveraging social interactions; that is, proximity to other scientists. Our analysis attempts to identify and quantify drivers of research portfolio diversification. Based on data concerning a very large sample of physicists we find that, while knowledge relatedness plays a role, contemporary science is a profoundly social enterprise. When scientists move out of their specialization, they do so through collaborations. And the further they move, the more these collaborations matter.

Limitations in the methodology we employed for this study point towards needed future developments. First and foremost, we are not assessing causal effects; we analyse research diversification patterns irrespective of the mechanisms which determine the similarity among sub-fields and the co-authorship network. Indeed, knowledge relatedness and collaborations may themselves be affected by scientists’ diversification strategies. We believe that the observed negative interaction between knowledge and social relatedness helps us rule out, at least partially, the contingency of reverse causality for social relatedness: if diversification were causally driving the link, we would expect a positive interaction. There is no reason to believe that new collaborators are easier to find in sub-fields far from a scientist’s own specialization; in fact, the opposite may be more likely—the closer the sub-fields, the higher the chances to collaborate. Moreover, since the structure of the knowledge space appears fairly stable over time, the direction of causality is more likely from subject proximity to diversification—not the other way around. Additional analyses with methods that fully exploit the temporal trajectories of scientists’ activities will be instrumental to elucidating the causal interplay between individual strategies and collaborations. In the Supplement we do provide results for the checks we were able to run based on the data and methods at our disposal.

Another critical development will be expanding the investigation to scientific and/or technological domains beyond physics—shedding further light on behaviours and potential sources of heterogeneity. Our initial focus on physics was due to its central role in the natural sciences and to the availability of reliable and abundant data. Nevertheless, the approach used in this study is fully applicable to different domains. Patents and publications records would both be useful grounds to validate and extend our results—thus providing a quantitative benchmark to inform science and technology policy.

From a policy perspective, our current results already provide some insights. They support the notion that social interactions constitute the core medium to foster new scientific venues, allowing scientists to overcome knowledge barriers. Thus, social interactions should be a focus of efforts aimed at improving cross-disciplinary team formation. Institutions should strive to create environments that favor social proximity and collaboration, and funding for interdisciplinary research should reward matches among scholars specialized in very distant domains.

## Methods

### Data

We use the American Physical Society (henceforth APS) dataset, which is maintained by the APS and publicly available for research purposes upon request (see the APS website). Each article in the dataset is labeled with up to 5 PACS codes. As an example, the PACS code *42.65.-k* refers to *nonlinear optics*; the first digit represents a broad field (Classical Physics), and the second a more specific sub-field (Optics). A brief description of the one-digit level fields is provided in Table S1. In our analyses, we work at the level of sub-fields; our measure of knowledge relatedness is based on similarity of PACS at two-digit level. Based on our aims (analysing research diversification strategies), we created a dataset based on two requirements: (i) the ability to reconstruct the career of each individual, and (ii) a standardized classification system for each article. (i) poses several issues related to name disambiguation, which have been successfully investigated in previous studies. We rely on the disambiguated dataset made available by Sinatra et al.^21^ (ii) concerns the classification scheme applied to physics articles. The PACS classification has been broadly employed from 1970 to 2016, but then the APS adopted a different labelling procedure (Physics Subject Headings; PhySH). We limit our analysis to a period entirely covered by the PACS system. Our final dataset includes information regarding 197,682 scholars that published at least one article in one of the 9 APS journals in the period ranging from 1977 to 2009. Figure S1 shows the number of papers (panel **a**) and the number of papers per author (panel **b**) over time.

### Monopartite projections of bipartite networks

A bipartite network is a graph whose nodes can be divided into two distinct sets (layers) such that no edge connects a pair of nodes belonging to the same set. A binary undirected bipartite network is identified by a rectangular biadjacency matrix **b** of dimensions $N_R \times N_C$. The number of rows $N_R$ is the number of nodes in layer *R*, and the number of columns $N_C$ is the number of nodes in layer *C*^22^. Being binary simply means that the elements of the matrix are

2 $$\begin{aligned} b_{_{rc}}={\left\{ \begin{array}{ll} 1 &{} \text {if node } \textit{r} \in \text { R and } \textit{c} \in \text { C are linked}\\ 0 &{} \text {otherwise} \end{array}\right. } \end{aligned}$$

The weighted monopartite projection on one of the layers is constructed counting so-called V-motifis: we draw a link in the projected network if two nodes share a neighbour in the bipartite network. For instance, to derive the weighted monopartite projection on layer R, we count co-occurences in the bipartite network and construct the square $N_R \times N_R$ matrix **M** with elements

3 $$\begin{aligned} m_{rr'}=\sum _{c=1}^{N_C}b_{rc}b_{r'c} \end{aligned}$$

For our analyses, we derive weighted monopartite projections from three binary bipartite networks; namely, Subfields-Articles, Authors-Articles and Subfields-Authors.

### Scientists’ specializations

Our analyses require us to assign specializations (single or multiple) to individuals. Unfortunately, there is no standard way to approach this problem—in part because, unlike articles or patents which can often be unambiguously linked to a limited number of classes, scientists can explore the knowledge space quite extensively. For our purposes, a suitable assignment should take into account both the relative specialization of a scientist and the distribution of publications across areas. Share-based metrics can be used to construct effective assignments. An instance is the Revealed Scientific Advantage (RSA) recently used by Battiston et al.^7^, which is akin to a metric originally used by Balassa^23^ to analyse comparative international trade advantages among countries. We consider the normalized metric; for each author *i* and sub-field (two-digit PACS) *s* this is defined as

4 $$\begin{aligned} RSA_{is} = \frac{\frac{w_{i,s}}{\sum _s w_{i,s}} }{\frac{\sum _i w_{i,s}}{\sum _{i,s} w_{i,s}}}, \end{aligned}$$

where $w_{i,s}$ is the number of articles author *i* has published in sub-field *s*. By construction, $RSA_{is} \in [-1,1]$, and a positive value indicates an advantage for author *i* in sub-field *s*. To assign a single specialization to *i*, we simply take $s(i) = argmax_s \lbrace RSA_{is} \rbrace$.

To assign multiple specializations to *i*, we take $S(i) = \{s\ s.t.\ RSA_{is}>0\}$. In this case we actually create a fictitious “copy” of *i* for each of the sub-fields in *S*(*i*)—keeping all individual characteristics but the specialization for each copy. This overcomes possible biases stemming from classification errors or marked heterogeneity in the distribution of articles across sub-fields.

### Measures of knowledge and social relatedness

We define knowledge relatedness among sub-fields (two-digit PACS) from the bipartite network PACS-Articles. Specifically, we derive the monopartite projection on the PACS layer (a $68 \times 68$ co-occurrence matrix) and then apply the cosine similarity to construct a knowledge relatedness matrix. The procedure is illustrated in Figure 3: panel **a** shows a stylized example of the bipartite network PACS-Articles, panel **b** shows the network of co-occurrences of all pairs of PACS (the monopartite projection on the PACS layer), and panel **c** shows the cosine similarity matrix describing proximity among physics sub-fields.

We define social relatedness from the initial co-authorship network *G*(*V*, *E*). Specifically, we build an augmented graph $G'(V',E')$ to integrate scientists’ specializations: for each node (author) $V \in G$, we create an *individual* node in $G'$ and for each edge $E\in G$ we draw the corresponding edge in $G'$. Then for each PACS *s*, we create an *attribute* node in $G'$. Next, we add further edges to $G'$ considering the specialization(s) of each scientist and creating an edge between her individual node and her specialization(s)’s attribute node(s) (Fig. 3**d** provides a simple example). Finally, we capture social relatedness with a binary variable based on whether an author has at least one coauthor specialized in a sub-field different from her own; that is

5 $$\begin{aligned} SR_{is}= {\left\{ \begin{array}{ll} 1 &{} \text {if}\ d(i,s)=2\\ 0 &{} \text {otherwise} \end{array}\right. } \end{aligned}$$

where *d*(*i*, *s*) is the geodesic distance between scientist *i* and sub-field *s* in the augmented graph.

### Modeling and assessment of predictors’ contributions

Consider an author *i* specialized in the sub-field *a*. The probability that she is also active in sub-field $b\ne a$ is modeled as

6 $$\begin{aligned} p:= f(KR_{ab},SR_{ib},\mathbf {IF}_\mathbf {i},\mathbf {SC}_\mathbf {b},\mathbf {Cit}_\mathbf {b}) \end{aligned}$$

where $KR_{ab}$ is the knowledge relatedness between the two sub-fields, $SR_{ib}$ is the social relatedness between the author and the sub-field *b*, $\mathbf {IF}_{\mathbf {i}}$ is a vector of author’s characteristics, $\mathbf {SC}_{\mathbf {b}}$ is a vector of variables capturing the sub-field popularity and competition (i.e., for each sub-field, number of papers and number of specialized scientists), and $\mathbf {Cit}_{\mathbf {b}}$ is a vector of variables capturing the relative attractiveness of the sub-field. A full list of the variables comprised in these vectors is provided in Table S3. We reformulate the model as a logistic regression and consider two baseline specifications, with and without the interaction term between knowledge and social relatedness:

7 $$\begin{aligned} ln\left(\frac{p}{1-p}\right) = \alpha + \beta KR_{ab} + \gamma SR_{ib} + \varvec{\theta } \cdot \mathbf {IF}_{\mathbf {i}} + \varvec{\eta } \cdot \mathbf {SC}_{\mathbf {b}} + \varvec{\phi } \cdot \mathbf {Cit}_{\mathbf {b}} \end{aligned}$$

8 $$\scriptsize{\begin{aligned} ln \left (\frac{p}{1-p} \right) = \alpha + \beta KR_{ab} + \gamma SR_{ib} + \zeta (KR_{ab} \times SR_{ib}) + \varvec{\theta} \cdot \mathbf {IF}_{\mathbf {i}} + \varvec{\eta } \cdot \mathbf {SC}_{\mathbf {b}} + \varvec{\phi} \cdot \mathbf {Cit}_{\mathbf {b}} \end{aligned}}$$

For both the single-and multiple-specialization settings, we fit these logistic regressions in three scenarios; namely, *full* (no constraint on sub-fields *a* and *b*), *within field* (*a* and *b* in the same field; i.e. one-digit PACS code) and *between field* (*a* and *b* in different fields) diversification.

In order to quantify the roles of knowledge and social relatedness, we compute the *Relative Contribution to Deviance Explained* (RCDE) for each of these variables^24^. For a generic predictor *X* this is defined as

9 $$\begin{aligned} RCDE_{X} = \frac{(D_{null}-D_{full}) - (D_{null}-D_{full \setminus X})}{(D_{null}-D_{full})} \end{aligned}$$

where $D_{null}$ is the null deviance, $D_{full}$ is the residual deviance of the full model (including all predictors) and $D_{full \setminus X}$ is the residual deviance of the model obtained by removing *X* (in our case *KR* or *SR*). The RCDE thus quantifies the percentage of the total logistic deviance attributable *X*.

## Supplementary information

Supplementary Information 1.

## Data Availability

All data used in this study are publicly available. The APS data can be downloaded at https://journals.aps.org/datasets. The list of disambiguated authors’ names is available at https://science.sciencemag.org/content/354/6312/aaf5239.

## Code availability

Code and functions for regression and other statistical analyses/tests are available in standard R libraries and packages including cooccur, stats (glm), interplot, sandwich. Other code used in this study (e.g., for data cleaning and results visualization, based on Python and R packages) is available from the corresponding authors upon request.

## References

[1] Kuhn, T. S. & Epstein, J. *The Essential Tension: Selected Studies in Scientific Tradition and Change*. (University of Chicago Press, 1977).

[2] Jones BF (2009). The burden of knowledge and the “death of the renaissance man”: Is innovation getting harder?. Rev. Econ. Stud..

[3] Wuchty S, Jones BF, Uzzi B (2007). The increasing dominance of teams in production of knowledge. Science.

[4] Foster JG, Rzhetsky A, Evans JA (2015). Tradition and innovation in scientists’ research strategies. Am. Sociol. Rev..

[5] Pan RK, Sinha S, Kaski K, Saramäki J (2012). The evolution of interdisciplinarity in physics research. Sci. Rep..

[6] Sun, Y. & Latora, V. The evolution of knowledge within and across fields in modern physics. *Sci Rep* **10**, 12097 (2020). 10.1038/s41598-020-68774-w PMC7374558 32694516

[7] Battiston F (2019). Taking census of physics. Nat. Rev. Phys..

[8] Aleta A, Meloni S, Perra N, Moreno Y (2019). Explore with caution: Mapping the evolution of scientific interest in physics. EPJ Data Sci..

[9] Jia T, Wang D, Szymanski BK (2017). Quantifying patterns of research-interest evolution. Nat. Hum. Behav..

[10] Zeng A (2019). Increasing trend of scientists to switch between topics. Nat. Commun..

[11] Breschi S, Lissoni F, Malerba F (2003). Knowledge-relatedness in firm technological diversification. Res. Policy.

[12] Teece DJ, Rumelt R, Dosi G, Winter S (1994). Understanding corporate coherence: Theory and evidence. J. Econ. Behav. Organ..

[13] Veech JA (2013). A probabilistic model for analysing species co-occurrence. Glob. Ecol. Biogeogr..

[14] Tumminello M, Micciche S, Lillo F, Piilo J, Mantegna RN (2011). Statistically validated networks in bipartite complex systems. PLoS ONE.

[15] Bowen Y, Jianxi L (2016). Measuring technological distance for patent mapping. J. Assoc. Inf. Sci. Technol..

[16] Lamperti, F., Malerba, F., Mavilia, R. & Tripodi, G. Does the position in the inter-sectoral knowledge space affect the international competitiveness of industries?. *Econ. Innov. New Technol. ***29,** 441–488 (2020).

[17] Zeileis A (2004). Econometric computing with hc and hac covariance matrix estimators. J. Stat. Softw..

[18] Yuan M, Lin Y (2006). Model selection and estimation in regression with grouped variables. J. R. Stat. Soc. Ser. B Stat. Methodol..

[19] Mullainathan S, Spiess J (2017). Machine learning: An applied econometric approach. J. Econ. Perspect..

[20] Chinazzi M, Gonçalves B, Zhang Q, Vespignani A (2019). Mapping the physics research space: A machine learning approach. EPJ Data Sci..

[21] Sinatra R, Wang D, Deville P, Song C, Barabási A-L (2016). Quantifying the evolution of individual scientific impact. Science.

[22] Saracco F (2017). Inferring monopartite projections of bipartite networks: An entropy-based approach. New J. Phys..

[23] Balassa B (1965). Trade liberalisation and “revealed” comparative advantage 1. Manchester Sch..

[24] Campos-Sánchez R, Cremona MA, Pini A, Chiaromonte F, Makova KD (2016). Integration and fixation preferences of human and mouse endogenous retroviruses uncovered with functional data analysis. PLoS Comput. Biol..

