# Supplementary material for: Knowledge and social relatedness shape research portfolio diversification
Source: Sci Rep. 2020 Aug 28;10:14232. doi: 10.1038/s41598-020-71009-7 (PMC7455750; doi:10.1038/s41598-020-71009-7)
Supplement: Supplementary file 1 — Supplementary Information 1. [file 41598_2020_71009_MOESM1_ESM.pdf]

Supplementary Information

Knowledge and Social Relatedness Shape Research Portfolio Diversification

Giorgio Tripodi[1,*], Francesca Chiaromonte[2,3,*], and Fabrizio Lillo[1,4,*]

[1]Scuola Normale Superiore, Pisa, Italy

[2]Institute of Economics and EMbeDS, Scuola Superiore Sant'Anna Pisa, 56127 Pisa, Italy

[3]Department of Statistics, The Pennsylvania State University, University Park, PA 16802

[4]Department of Mathematics, University of Bologna, 40126 Bologna, Italy

[*]Correspondence to: Giorgio Tripodi, giorgio.tripodi@sns.it; Francesca Chiaromonte, fxc11@psu.edu; Fabrizio Lillo, fabrizio.lillo@unibo.it

Supplementary Information

S1 Data

The American Physical Society (APS) grants access to data containing information about papers published in 9 journals: Physical Review A, B, C, D, E, I, L, ST and Review of Modern Physics. The APS makes available, under request, two datasets including over 450,000 articles metadata and citations from 1893 onwards. Each article has a unique identifier and most of them contain reference codes that map into physics sub-fields (PACS codes). As mentioned in section 4.1, we make use of such a classification to keep track of scientists' diversification patterns. Moreover, we use a disambiguated list of authors made available by [1]. As a result, we analyse a sub-sample for which we have access to all the necessary information: it includes more than 300,000 articles published by 197,682 authors over the period 1977-2009. Figure S1 provides simple statistical properties of the dataset.

[Figure S1 about here.]

[Table S1 about here.]

S2 Test of randomness - multiple hypothesis correction

As stated in section 2.2, under the hypothesis that scientists diversify their research portfolio at random, the probability that exactly x authors are active in two sub-fields follows a hypergeometric distribution [2]. A clear advantage of such a formulation is that we can easily associate a p-value to each element (i.e., link in the projected network) and evaluate significance. However, since we are performing hypothesis testing, we need to set a level of statistical significance accordingly. One of the most commonly used method to deal with multiple hypothesis testing is the *Bonferroni* correction. Such a well known method controls for *family-wise error rate* (FWER) in a very stringent fashion, computing the adjusted p-value by directly multiplying the number of simultaneously tested hypotheses. In many multiple testing settings, the Bonferroni correction might result too strict, and not appropriate in dealing with dependence. Thus, many less restrictive and more flexible alternatives such as FDR have been developed [3, 4]. Table S2 reports results for non-corrected p-values and after several correction methods:

Bonferroni, Benjamini-Hochberg (BH), and Benjamini-Yekutieli (BY). Results confirm that scientists' diversification choices can be hardly seen as a random phenomenon, irrespective of the correction method employed.

[Table S2 about here.]

S3 Additional estimation results

As mentioned in section 4.5 and 2.3, we use a multivariate logistic regression to estimate the probability that a scientist diversifies in a sub-field different from her own specialization. Table S3 summarizes our independent variables and includes information about our grouping strategy. Here, we provide results for each and every specification: (i) single specialization (full diversification), (ii) multiple-specialization (full diversification), (iii) single specialization (within field diversification), (iv) multiple specialization (within field diversification), (v) single specialization (between field diversification) and (vi) multiple specialization (between field diversification).

[Table S3 about here.]

Full diversification - Specification (i) and (ii) Results are summarized in Table S4 and S5, where the first column refers to the baseline model (without the interaction term between social and knowledge relatedness), column (2) refers to the model including the interaction term while column (3) presents the same results with clustering corrected standard errors. Figure 5-**a**/**c** show the differences in the probability of diversification as a function of knowledge and social relatedness, taking into account all the control variables. Figure 5-**b**/**d** provide evidence of the moderating role played by the similarity across sub-fields on the estimated coefficient of social relatedness

[Table S4 about here.]

[Table S5 about here.]

Within field diversification - Specification (iii) and (iv). Figure S2-**a**/**b** plots the results for the single specialization case: knowledge and social relatedness are still significant as well

as their interaction, but the magnitude of the coefficients is smaller with respect to the full diversification case. In addition, when we consider the multiple specialization case (Figure S2-**c/d**), coefficients shrink further and the interaction term between social and knowledge relatedness is no longer significant (see Table S6 and S7 for details).

[Figure S2 about here.]

[Table S6 about here.]

[Table S7 about here.]

Between field diversification - Specification (v) and (vi). As far as the between field diversification is concerned, the general trends in terms of social and cognitive proximity are confirmed. Moreover, the negative interaction term remains statistically significant and not negligible in magnitude for both model specifications (single and multiple specialization). Figure S3, Table S8 and Table S9 summarize the results.

[Figure S3 about here.]

[Table S8 about here.]

[Table S9 about here.]

S4 Temporal evolution of the physics knowledge space

The structure of the knowledge space can evolve over time, and sharp differences might undermine our strategy. To check whether such changes are significant, we split our initial dataset into three subsets, one for each decade: 1980-1989, 1990-1990, 2000-2009. We compare the structure of the physics knowledge space in the last decade of our sample with the one referring to the entire period. Figure S4 compares popularity of one- and two-digit PACS in the last decade with the one for the full sample. Figure S5 shows how the network and, as a consequence, the cosine similarity matrix have changed in the last ten years. Figure S6 shows the popularity of one- and two-digit PACS in the three decades. Data confirm the rise of interdisciplinary physics within an otherwise stable distribution of interests, as much as it was observed in previous works [5].

[Figure S4 about here.]

[Figure S5 about here.]

[Figure S6 about here.]

Since our measure of knowledge relatedness depends on PACS co-occurrences in research articles, we provide a more robust quantitative test to check whether the relationships among sub-fields have changed significantly over time. To do so, we first construct the difference between the cosine similarity matrix in two decades (see Figure S7 and S8). Then we validate the resulting difference matrices against the null of zero difference by sampling with replacement and generating 1,000 additional of such matrices. Finally, we compute the confidence interval ($\alpha = 0.05$) for each element of the difference matrix to assess its statistical significance, taking into account multiple hypothesis testing issues (Bonferroni correction). Figure S9 shows the results of the bootstrap validation procedure (statistically significant pairs in black). In general, the number of significant element is not large, especially for consecutive decades, indicating a fairly stable structure of the physics knowledge space. More importantly, the analysis discussed in Section S5.2, where past knowledge space is used in the regression, shows that changes in knowledge relatedness do not affect the main conclusions on the drivers of research portfolio diversification.

[Figure S7 about here.]

[Figure S8 about here.]

[Figure S9 about here.]

S5 Alternative estimation strategies

S5.1 Multidisciplinarity

Keeping track of diversification patterns for truly multidisciplinary scientists is a non-trivial task. Indeed, some scientists might have several core specializations leading to a positive bias in the previous estimates. To take into account this issue, we present an additional robustness check to validate further our empirical strategy: we assign each scientist to a single specialization

- the one corresponding to the maximum value of RSA - but we constrain the choices of each scientists by eliminating from the regression the possibility to diversify in any of the PACS for which $RSA > 0$. In other words, we take into account only truly unexplored sub-fields. Figure S10 confirms that scientists research portfolio diversification depends on social and knowledge relatedness, and the two measures interact with each other. Table S10 summarizes the results.

[Figure S10 about here.]

[Table S10 about here.]

S5.2 Time dimension

The temporal dimension is of paramount importance when evaluating scientific activities, especially to disentangle the direction of causality. Over time, our measures of knowledge and social relatedness might be affected by scientists' research diversification themselves. We tackle this issue by running an additional robustness check to isolate the effect of our measures on scientists' diversification strategies. First, we split our dataset into three time periods (i.e., three decades: 1980-1989, 1990-1990, 2000-2009) and we identify 15,466 scientists active in all periods. Then, we compute our measures of knowledge and social relatedness for each period to predict authors' diversification in a given decade using relatedness measures of a past decade. As before, we use a logistic regression where our dependent variable is a binary one (being active in a sub-field different from specialization), but this time we use knowledge and social relatedness computed at time $t-1$ and $t-2$. Formally, we use three econometric specifications:

$$Y_{t-1} = \alpha + \beta KR_{t-2} + \gamma SR_{t-2} \ + \zeta(KR_{t-2} \times SR_{t-2}) + \delta field\ core + \epsilon \tag{1}$$

$$Y_{t} = \alpha + \beta KR_{t-1} + \gamma SR_{t-1} \ + \zeta(KR_{t-1} \times SR_{t-1}) + \delta field\ core + \epsilon \tag{2}$$

$$Y_{t} = \alpha + \beta KR_{t-2} + \gamma SR_{t-2} \ + \zeta(KR_{t-2} \times SR_{t-2}) + \delta field\ core + \epsilon \tag{3}$$

where t indicates the last decade (2000-2009). Such additional tests provide indication of the direction of causality since we take in account social and cognitive proximity prior to the scientists' choice to diversify. Moreover, we only consider sub-fields never explored before by each author so to approximate a quasi-experimental setting. Results confirm the role played by knowledge

and social relatedness as well as the negative interaction between our two measures (see Figure S11 and Table S11).

[Figure S11 about here.]

[Table S11 about here.]

References

[1] Roberta Sinatra, Dashun Wang, Pierre Deville, Chaoming Song, and Albert-László Barabási. Quantifying the evolution of individual scientific impact. *Science*, 354(6312), 2016.

[2] Michele Tumminello, Salvatore Micciche, Fabrizio Lillo, Jyrki Piilo, and Rosario N Mantegna. Statistically validated networks in bipartite complex systems. *PloS one*, 6(3):e17994, 2011.

[3] Yoav Benjamini and Yosef Hochberg. Controlling the false discovery rate: a practical and powerful approach to multiple testing. *Journal of the Royal statistical society: series B (Methodological)*, 57(1):289–300, 1995.

[4] Yoav Benjamini and Daniel Yekutieli. The control of the false discovery rate in multiple testing under dependency. *Annals of statistics*, pages 1165–1188, 2001.

[5] Raj Kumar Pan, Sitabhra Sinha, Kimmo Kaski, and Jari Saramäki. The evolution of interdisciplinarity in physics research. *Scientific reports*, 2:551, 2012.

Table S1: One-digit PACS codes

PACS	Field	Description
0	General	Mathematical Methods, Quantum Mechanics, Relativity, Nonlinear Dynamics and Metrolog
1	High-energy	Physics of Elementary Particles and Fields
2	Nuclear	Nuclear Structure and Reactions
3	Atomic	Atomic and Molecular Physics
4	Classical	Electromagnetism, Optics, Acoustics, Heat Transfer, Classical Mechanics and Fluid Dynamics
5	Plasma	Physics of Gases, Plasmas and Electric Discharges
6 - 7	Condensed Matter	Structural, Mechanical and Thermal Properties, Electronic Structure and Electrical, Magnetic and Optical Properties
8	Interdisc	Interdisciplinary Physics and Related Areas of Science and Technology
9	Astro	Astrophysics, Astronomy and Geophysics

Table S2: Test of randomness in scientists' research portfolio diversification

	**Positive**	**Negative**	**% Non-Random**
**No correction**	1361	616	86.8
**Bonferroni**	1151	486	71.8
**BH**	1339	580	84.2
**BY**	1264	547	79.4

Note: 2,278 pairs analyzed, 68 sub-fields, 197,682 scientists. Analysis performed employing the **R** package *cooccur*.

Table S3: Variables and grouping strategy

Name	Group	Description
Knowledge relatedness	1 - KR	Cosine similarity among sub-fields
Social relatedness	2 - SR	Scientist' co-authors specialized in the sub-field different from her core one (dummy)
Field core	3 - IF	macro-field specialization (categorical)
# of PACS	4 - IF	Number of PACS explored
# of papers	4 - IF	Number of papers published
# of co-authors	4 - IF	Number of co-authors
PACS target popularity	5 - SC	Number of articles assigned to the target sub-field
Δ crowd	5 - SC	Difference in the number of specialized scientists between core and target sub-field
Δ PACS citations	6 - Cit	Difference in the number citations between core and target sub-field
Δ field citations	6 - Cit	Difference in the number citations between core and target macro-field

Table S4: (i) Single specialization - full diversification.

	Dependent variable: P(diversification)		
	Baseline	Interactions	Robust SE
	(1)	(2)	(3)
Knowledge Relatedness	0.846***	0.936***	0.936***
	(0.002)	(0.002)	(0.003)
Social Relatedness	2.647***	2.827***	2.827***
	(0.004)	(0.005)	(0.006)
field core-Atomic	−0.332***	−0.332***	−0.332***
	(0.009)	(0.009)	(0.010)
field core-Classical	−0.480***	−0.490***	−0.490***
	(0.010)	(0.010)	(0.010)
field core-Cond.matter	−1.094***	−1.088***	−1.088***
	(0.010)	(0.010)	(0.012)
field core-General	−0.710***	−0.722***	−0.722***
	(0.010)	(0.010)	(0.011)
field core-High.energy	0.221***	0.219***	0.219***
	(0.011)	(0.011)	(0.010)
field core-Interdisc	−0.546***	−0.557***	−0.557***
	(0.009)	(0.009)	(0.010)
field core-Nuclear	0.438***	0.463***	0.463***
	(0.009)	(0.009)	(0.010)
field core-Plasma	−0.258***	−0.269***	−0.269***
	(0.014)	(0.014)	(0.013)
# of PACS	0.891***	0.882***	0.882***
	(0.003)	(0.003)	(0.002)
# of papers	−0.007**	0.010***	0.010***
	(0.003)	(0.003)	(0.002)
PACS target popularity	1.130***	1.130***	1.130***
	(0.003)	(0.003)	(0.002)
Δ crowd	0.239***	0.239***	0.239***
	(0.002)	(0.002)	(0.002)
# of co-authors	−0.382***	−0.406***	−0.406***
	(0.003)	(0.003)	(0.002)
Δ PACS citations	−0.272***	−0.273***	−0.273***
	(0.003)	(0.003)	(0.002)
Δ field citations	−0.167***	−0.156***	−0.156***
	(0.003)	(0.003)	(0.004)
KR:SR		−0.255***	−0.255***
		(0.004)	(0.004)
Constant	−3.749***	−3.812***	−3.812***
	(0.008)	(0.008)	(0.010)
Observations	7,072,386	7,072,386	7,072,386
Log Likelihood	−1,088,731.000	−1,086,281.000	−1,086,281.000
Akaike Inf. Crit.	2,177,498.000	2,172,600.000	2,172,600.000
Note:			*p<0.1; **p<0.05; ***p<0.01

Table S5: (ii) Multiple-specialization - full diversification

	Dependent variable:		
	Y		
	Baseline	Interactions	Robust SE
	(1)	(2)	(3)
Knowledge Relatedness	0.628***	0.689***	0.689***
	(0.001)	(0.005)	(0.009)
Social Relatedness	4.221***	4.243***	4.243***
	(0.005)	(0.005)	(0.019)
field core-Atomic	−0.427***	−0.427***	−0.427***
	(0.004)	(0.004)	(0.007)
field core-Classical	−0.475***	−0.475***	−0.475***
	(0.005)	(0.005)	(0.007)
field core-Cond.matter	−0.761***	−0.760***	−0.760***
	(0.004)	(0.004)	(0.009)
field core-General	−0.537***	−0.537***	−0.537***
	(0.004)	(0.004)	(0.007)
field core-High.energy	0.165***	0.165***	0.165***
	(0.005)	(0.005)	(0.006)
field core-Interdisc	−0.552***	−0.552***	−0.552***
	(0.004)	(0.004)	(0.007)
field core-Nuclear	0.163***	0.163***	0.163***
	(0.004)	(0.004)	(0.006)
field core-Plasma	−0.409***	−0.409***	−0.409***
	(0.007)	(0.007)	(0.008)
# of PACS	0.768***	0.768***	0.768***
	(0.001)	(0.001)	(0.003)
# of papers	0.117***	0.117***	0.117***
	(0.001)	(0.001)	(0.003)
PACS target popularity	0.611***	0.611***	0.611***
	(0.001)	(0.001)	(0.002)
Δ crowd	0.359***	0.359***	0.359***
	(0.001)	(0.001)	(0.003)
# of co-authors	−0.346***	−0.346***	−0.346***
	(0.001)	(0.001)	(0.004)
Δ PACS citations	−0.333***	−0.333***	−0.333***
	(0.001)	(0.001)	(0.003)
Δ field citations	−0.071***	−0.070***	−0.070***
	(0.001)	(0.001)	(0.004)
KR:SR		−0.062***	−0.062***
		(0.005)	(0.010)
Constant	−5.855***	−5.877***	−5.877***
	(0.006)	(0.007)	(0.020)
Observations	35,562,394	35,562,394	35,562,394
Log Likelihood	−7,299,777.000	−7,299,692.000	−7,299,692.000
Akaike Inf. Crit.	14,599,590.000	14,599,421.000	14,599,421.000
Note:			*p<0.1; **p<0.05; ***p<0.01

Table S6: (iii) Single specialization - within field diversification

	Dependent variable:		
	Y		
	Baseline	Interactions	Robust SE
	(1)	(2)	(3)
Knowledge Relatedness	0.166***	0.184***	0.184***
	(0.004)	(0.004)	(0.005)
Social Relatedness	2.265***	2.272***	2.272***
	(0.008)	(0.008)	(0.008)
field core-Atomic	0.059***	0.056**	0.056**
	(0.022)	(0.022)	(0.025)
field core-Classical	−1.003***	−1.001***	−1.001***
	(0.026)	(0.026)	(0.029)
field core-Cond.matter	−1.108***	−1.110***	−1.110***
	(0.021)	(0.021)	(0.024)
field core-General	−0.931***	−0.927***	−0.927***
	(0.026)	(0.026)	(0.028)
field core-High.energy	1.809***	1.806***	1.806***
	(0.025)	(0.025)	(0.027)
field core-Interdisc	−0.353***	−0.357***	−0.357***
	(0.024)	(0.024)	(0.026)
field core-Nuclear	0.978***	0.969***	0.969***
	(0.021)	(0.021)	(0.024)
field core-Plasma	−0.149**	−0.155**	−0.155**
	(0.068)	(0.068)	(0.068)
# of PACS	0.769***	0.769***	0.769***
	(0.005)	(0.005)	(0.005)
# of papers	0.064***	0.065***	0.065***
	(0.006)	(0.006)	(0.005)
PACS target popularity	1.372***	1.370***	1.370***
	(0.006)	(0.006)	(0.005)
Δ crowd	0.130***	0.131***	0.131***
	(0.004)	(0.004)	(0.003)
# of co-authors	−0.239***	−0.240***	−0.240***
	(0.005)	(0.005)	(0.004)
Δ PACS citations	−0.209***	−0.208***	−0.208***
	(0.005)	(0.005)	(0.004)
KR:SR		−0.047***	−0.047***
		(0.007)	(0.007)
Constant	−1.883***	−1.882***	−1.882***
	(0.020)	(0.020)	(0.022)
Observations	1,000,230	1,000,230	1,000,230
Log Likelihood	−334,720.800	−334,697.300	−334,697.300
Akaike Inf. Crit.	669,475.700	669,430.600	669,430.600
Note:			*p<0.1; **p<0.05; ***p<0.01

Table S7: (iv) Multiple specialization - within field diversification

	Dependent variable:		
	Y		
	Baseline	Interactions	Robust SE
	(1)	(2)	(3)
Knowledge Relatedness	0.121***	0.121***	0.121***
	(0.009)	(0.009)	(0.013)
Social Relatedness	3.968***	3.968***	3.968***
	(0.010)	(0.010)	(0.021)
field core-Atomic	−0.276***	−0.276***	−0.276***
	(0.011)	(0.011)	(0.021)
field core-Classical	−0.932***	−0.932***	−0.932***
	(0.013)	(0.013)	(0.023)
field core-Cond.matter	−0.892***	−0.892***	−0.892***
	(0.011)	(0.011)	(0.020)
field core-General	−0.823***	−0.823***	−0.823***
	(0.012)	(0.012)	(0.021)
field core-High.energy	1.176***	1.176***	1.176***
	(0.012)	(0.012)	(0.023)
field core-Interdisc	−0.724***	−0.724***	−0.724***
	(0.012)	(0.012)	(0.021)
field core-Nuclear	0.692***	0.692***	0.692***
	(0.011)	(0.011)	(0.021)
field core-Plasma	−0.361***	−0.361***	−0.361***
	(0.041)	(0.041)	(0.058)
# of PACS	0.497***	0.497***	0.497***
	(0.002)	(0.002)	(0.004)
# of papers	0.252***	0.252***	0.252***
	(0.002)	(0.002)	(0.004)
PACS target popularity	0.774***	0.774***	0.774***
	(0.002)	(0.002)	(0.003)
Δ crowd	0.345***	0.345***	0.345***
	(0.002)	(0.002)	(0.003)
# of co-authors	−0.145***	−0.145***	−0.145***
	(0.002)	(0.002)	(0.004)
Δ PACS citations	−0.313***	−0.313***	−0.313***
	(0.002)	(0.002)	(0.003)
KR:SR	−0.001	−0.001	−0.001
	(0.009)	(0.009)	(0.013)
Constant	−4.250***	−4.250***	−4.250***
	(0.014)	(0.014)	(0.028)
Observations	5,407,404	5,407,404	5,407,404
Log Likelihood	−2,166,803.000	−2,166,803.000	−2,166,803.000
Akaike Inf. Crit.	4,333,642.000	4,333,642.000	4,333,642.000
Note:			*p<0.1; **p<0.05; ***p<0.01

Table S8: (v) Single specialization - between field diversification

	Dependent variable:		
	Y		
	Baseline	Interactions	Robust SE
	(1)	(2)	(3)
Knowledge Relatedness	0.622***	0.702***	0.702***
	(0.002)	(0.003)	(0.003)
Social Relatedness	2.768***	2.914***	2.914***
	(0.006)	(0.006)	(0.008)
field core-Atomic	−0.292***	−0.303***	−0.303***
	(0.010)	(0.010)	(0.010)
field core-Classical	−0.304***	−0.313***	−0.313***
	(0.010)	(0.010)	(0.010)
field core-Cond.matter	−1.294***	−1.263***	−1.263***
	(0.013)	(0.013)	(0.017)
field core-General	−0.628***	−0.632***	−0.632***
	(0.011)	(0.011)	(0.012)
field core-High.energy	−0.352***	−0.360***	−0.360***
	(0.013)	(0.014)	(0.013)
field core-Interdisc	−0.356***	−0.365***	−0.365***
	(0.010)	(0.010)	(0.011)
field core-Nuclear	0.060***	0.068***	0.068***
	(0.011)	(0.011)	(0.011)
field core-Plasma	−0.062***	−0.074***	−0.074***
	(0.014)	(0.014)	(0.015)
# of PACS	1.010***	1.003***	1.003***
	(0.004)	(0.004)	(0.004)
# of papers	−0.050***	−0.032***	−0.032***
	(0.004)	(0.004)	(0.004)
PACS target popularity	1.114***	1.108***	1.108***
	(0.003)	(0.003)	(0.003)
Δ crowd	0.322***	0.320***	0.320***
	(0.003)	(0.003)	(0.003)
# of co-authors	−0.461***	−0.488***	−0.488***
	(0.003)	(0.003)	(0.003)
Δ PACS citations	−0.375***	−0.369***	−0.369***
	(0.004)	(0.004)	(0.004)
Δ field citations	−0.209***	−0.196***	−0.196***
	(0.004)	(0.004)	(0.006)
KR:SR		−0.234***	−0.234***
		(0.004)	(0.005)
Constant	−4.115***	−4.168***	−4.168***
	(0.009)	(0.009)	(0.010)
Observations	6,072,156	6,072,156	6,072,156
Log Likelihood	−717,839.000	−716,398.900	−716,398.900
Akaike Inf. Crit.	1,435,714.000	1,432,836.000	1,432,836.000
Note:			*p<0.1; **p<0.05; ***p<0.01

Table S9: (vi) Multiple specialization - between field diversification

	Dependent variable:		
	Y		
	Baseline	Interactions	Robust SE
	(1)	(2)	(3)
cos	0.446***	0.511***	0.511***
	(0.001)	(0.005)	(0.011)
Social Relatedness	4.261***	4.284***	4.284***
	(0.006)	(0.006)	(0.021)
field core-Atomic	−0.384***	−0.385***	−0.385***
	(0.005)	(0.005)	(0.008)
field core-Classical	−0.328***	−0.328***	−0.328***
	(0.005)	(0.005)	(0.008)
field core-Cond.matter	−0.904***	−0.903***	−0.903***
	(0.005)	(0.005)	(0.013)
field core-General	−0.421***	−0.422***	−0.422***
	(0.005)	(0.005)	(0.008)
field core-High.energy	−0.060***	−0.060***	−0.060***
	(0.005)	(0.005)	(0.008)
field core-Interdisc	−0.367***	−0.367***	−0.367***
	(0.005)	(0.005)	(0.008)
field core-Nuclear	−0.160***	−0.161***	−0.161***
	(0.005)	(0.005)	(0.009)
field core-Plasma	−0.255***	−0.256***	−0.256***
	(0.007)	(0.007)	(0.009)
# of PACS	0.944***	0.944***	0.944***
	(0.001)	(0.001)	(0.004)
# of papers	0.050***	0.050***	0.050***
	(0.001)	(0.001)	(0.004)
PACS target popularity	0.559***	0.559***	0.559***
	(0.001)	(0.001)	(0.002)
Δ crowd	0.392***	0.393***	0.393***
	(0.002)	(0.002)	(0.003)
# of co-authors	−0.444***	−0.444***	−0.444***
	(0.001)	(0.001)	(0.006)
Δ PACS citations	−0.354***	−0.354***	−0.354***
	(0.002)	(0.002)	(0.003)
Δ field citations	−0.143***	−0.143***	−0.143***
	(0.002)	(0.002)	(0.005)
KR:SR		−0.067***	−0.067***
		(0.005)	(0.011)
Constant	−6.142***	−6.165***	−6.165***
	(0.007)	(0.008)	(0.022)
Observations	30,154,990	30,154,990	30,154,990
Log Likelihood	−4,971,576.000	−4,971,497.000	−4,971,497.000
Akaike Inf. Crit.	9,943,188.000	9,943,033.000	9,943,033.000
Note:			*p<0.1; **p<0.05; ***p<0.01

Table S10: Constrained diversification.

	Dependent variable:		
	Y		
	Baseline	Interactions	Robust SE
	(1)	(2)	(3)
Knowledge Relatedness	0.507***	0.586***	0.586***
	(0.005)	(0.006)	(0.007)
Social Relatedness	1.268***	1.398***	1.398***
	(0.012)	(0.013)	(0.014)
field core-Atomic	0.036	0.029	0.029
	(0.025)	(0.025)	(0.023)
field core-Classical	−0.034	−0.043*	−0.043*
	(0.026)	(0.026)	(0.024)
field core-Cond.matter	0.341***	0.342***	0.342***
	(0.027)	(0.027)	(0.028)
field core-General	−0.092***	−0.105***	−0.105***
	(0.027)	(0.027)	(0.026)
field core-High.energy	0.426***	0.418***	0.418***
	(0.030)	(0.030)	(0.027)
field core-Interdisc	0.040	0.023	0.023
	(0.026)	(0.026)	(0.024)
Nuclear	0.326***	0.341***	0.341***
	(0.027)	(0.027)	(0.024)
field core-Plasma	0.063*	0.058	0.058*
	(0.036)	(0.036)	(0.032)
# of PACS	0.761***	0.753***	0.753***
	(0.007)	(0.007)	(0.006)
# of papers	0.374***	0.389***	0.389***
	(0.007)	(0.007)	(0.006)
PACS target popularity	1.478***	1.473***	1.473***
	(0.006)	(0.006)	(0.006)
Δ crowd	0.193***	0.192***	0.192***
	(0.006)	(0.006)	(0.006)
# of co-authors	−0.099***	−0.116***	−0.116***
	(0.006)	(0.006)	(0.006)
Δ PACS citations	−0.380***	−0.381***	−0.381***
	(0.007)	(0.007)	(0.006)
Δ field citations	0.309***	0.320***	0.320***
	(0.008)	(0.008)	(0.009)
KR:SR		−0.230***	−0.230***
		(0.009)	(0.010)
Constant	−5.179***	−5.209***	−5.209***
	(0.024)	(0.024)	(0.024)
Observations	1,503,010	1,503,010	1,503,010
Log Likelihood	−165,560.600	−165,263.900	−165,263.900
Akaike Inf. Crit.	331,157.100	330,565.900	330,565.900
Note:			*p<0.1; **p<0.05; ***p<0.01

Table S11: Diversification (lag)

	Dependent variable:		
	Y_{t-1}	Y_t	
	lag1	lag1	lag2
	(1)	(2)	(3)
KR_{t-2}	0.030***		0.023***
	(0.0003)		(0.0003)
SR_{t-2}	1.165***		0.703***
	(0.037)		(0.047)
KR_{t-1}		0.023***	
		(0.0003)	
SR_{t-1}		0.941***	
		(0.035)	
field core-Atomic	−0.350***	−0.378***	−0.296***
	(0.043)	(0.053)	(0.054)
field core-Classical	−0.371***	−0.334***	−0.333***
	(0.047)	(0.058)	(0.059)
field core-Cond.matter	−0.495***	−0.434***	−0.383***
	(0.039)	(0.049)	(0.049)
field core-High.energy	−0.393***	−0.104*	−0.194***
	(0.046)	(0.057)	(0.057)
field core-Interdisc	−0.471***	−0.490***	−0.428***
	(0.046)	(0.060)	(0.061)
field core-Nuclear	−0.188***	−0.194***	−0.198***
	(0.042)	(0.053)	(0.054)
field core-Plasma	−0.416***	−0.395***	−0.319***
	(0.050)	(0.062)	(0.063)
$KR_{t-2} : SR_{t-2}$	−0.008***		−0.007***
	(0.001)		(0.001)
$KR_{t-1} : SR_{t-1}$		−0.005***	
		(0.001)	
Constant	−3.075***	−3.010***	−2.964***
	(0.038)	(0.047)	(0.048)
Observations	766,519	618,352	618,352
Log Likelihood	−180,229.100	−142,158.800	−143,114.400
Akaike Inf. Crit.	360,480.300	284,339.500	286,250.900
Note:			*p<0.1; **p<0.05; ***p<0.01

a

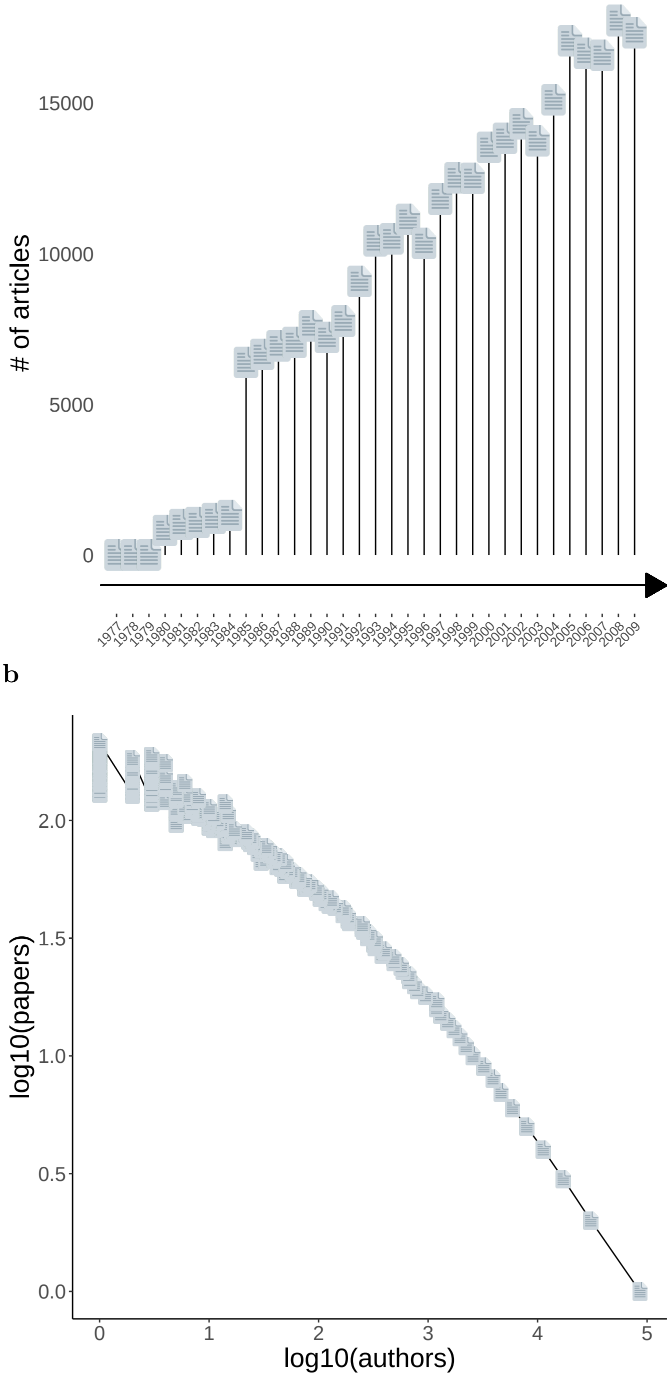

Figure S1: Statistical properties of the APS data. **a**, The time series of papers over time shows that the number of papers published in APS outlets increased substantially from 1977 to 2009. **b**, The distribution of the number of papers per author is fat-tailed: the large majority of authors published just few articles while some authors have been extremely productive.

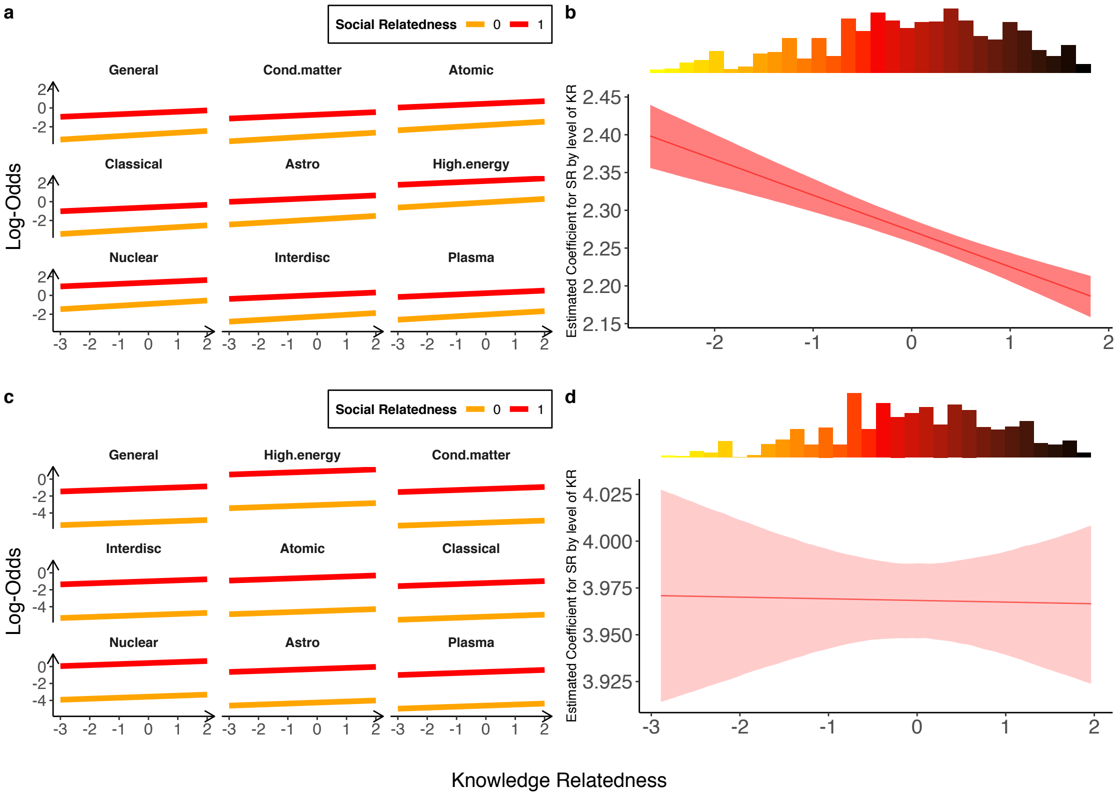

Figure S2: Scientists' research portfolio diversification - (within field diversification) single and multiple specialization. **a**, Log-odds as a function of social and (standardized) knowledge relatedness, controlling for all the confounding variables - specification (iii). **b**, Estimated coefficient for social relatedness conditional on (standardized) knowledge relatedness - specification (iii). **c**, Log-odds as a function of social and (standardized) knowledge relatedness, controlling for the all confounding variables - specification (iv). **d**, Estimated coefficient for social relatedness conditional on (standardized) knowledge relatedness - specification (iv). **b** and **d** include the distribution of the conditional variable (i.e., knowledge relatedness). The color palette is in accordance with the similarity matrix (Figure 3-**c**).

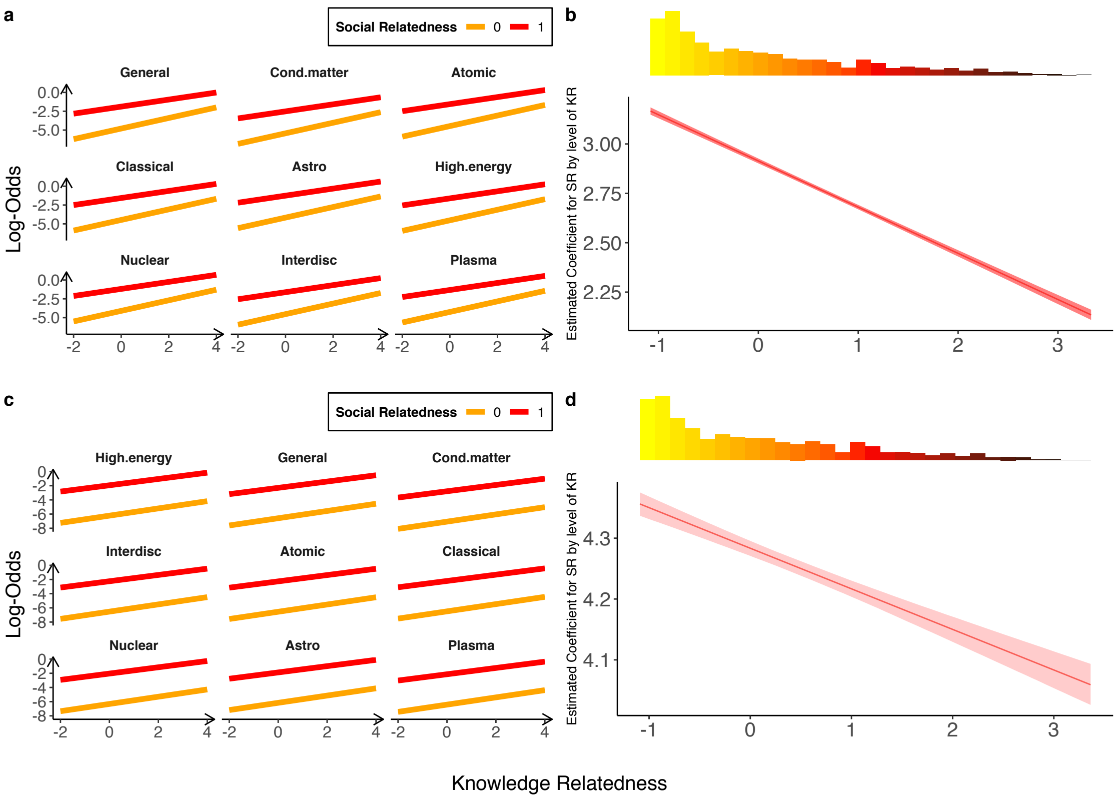

Figure S3: Scientists' research portfolio diversification - (between field diversification) single and multiple specialization. **a**, Log-odds as a function of social and (standardized) knowledge relatedness, controlling for all the confounding variables - specification (v). **b**, Estimated coefficient for social relatedness conditional on (standardized) knowledge relatedness - specification (v). **c**, Log-odds as a function of social and (standardized) knowledge relatedness, controlling for all the confounding variables - specification (vi). **d**, Estimated coefficient for social relatedness conditional on (standardized) knowledge relatedness - specification (vi). **b** and **d** include the distribution of the conditional variable (i.e., knowledge relatedness). The color palette is in accordance with the similarity matrix (Figure 3-**c**).

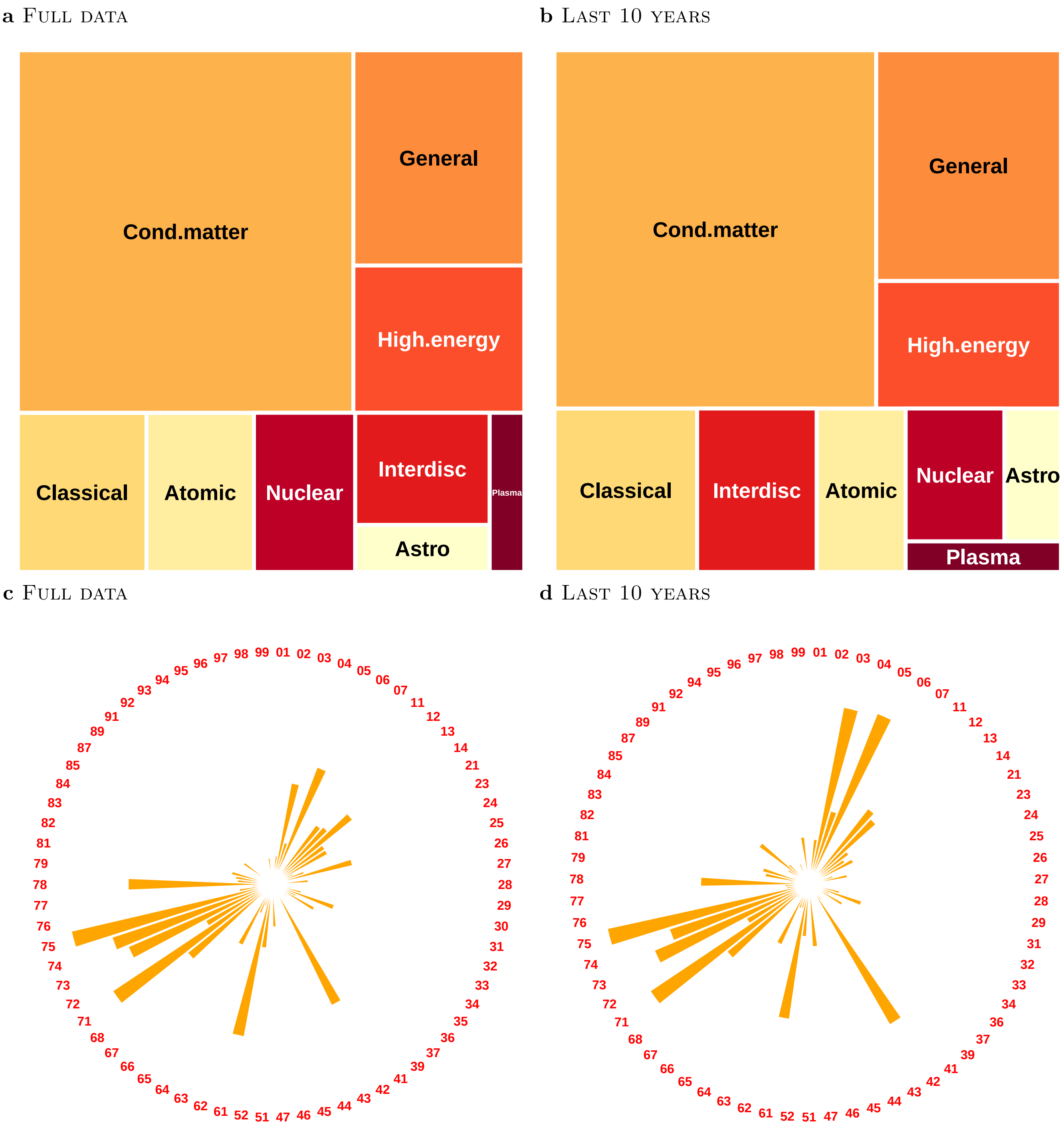

Figure S4: Popularity of fields and sub-fields over time. We focus on a subset including articles published from 2000 to 2009 (last 10 years in our data) to compare the popularity of physics fields and sub-fields over time (i.e., number of articles assigned to a given field/sub-field). The distribution of topics remains fairly stable, except for the rise of interdisciplinary physics.

a Full Data

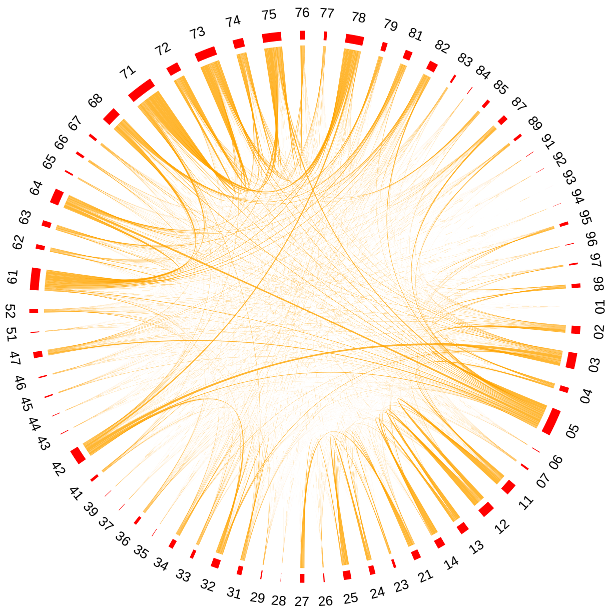

b Last 10 Years

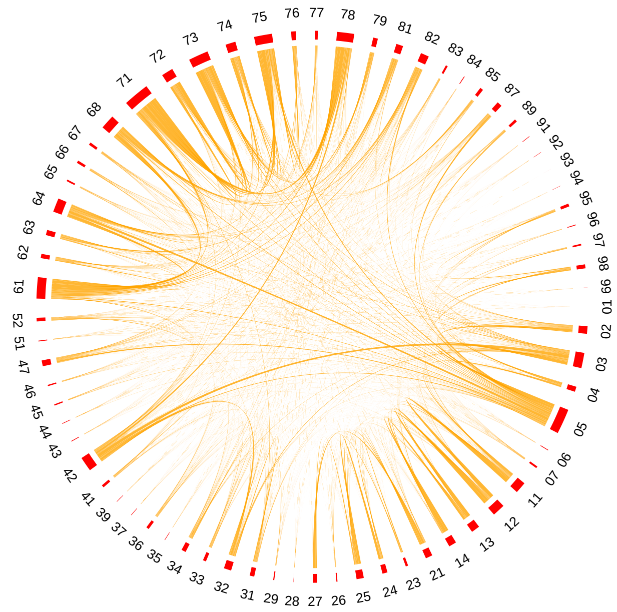

c Full Data

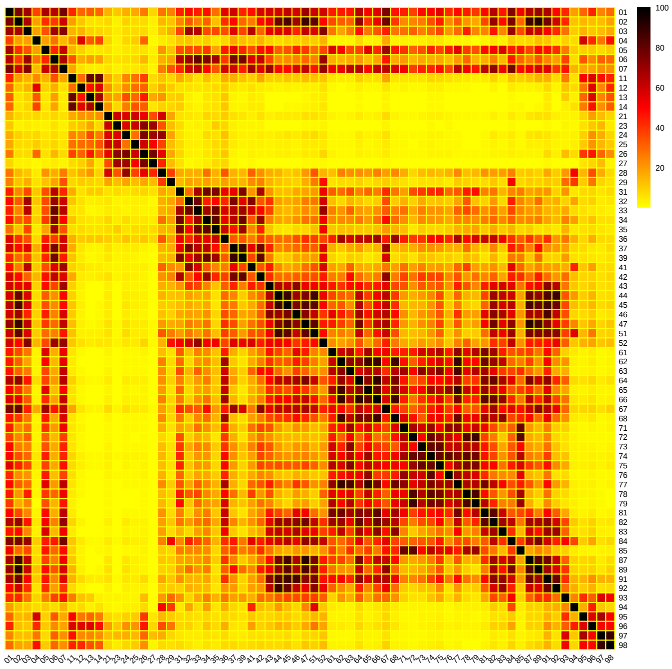

d Last 10 Years

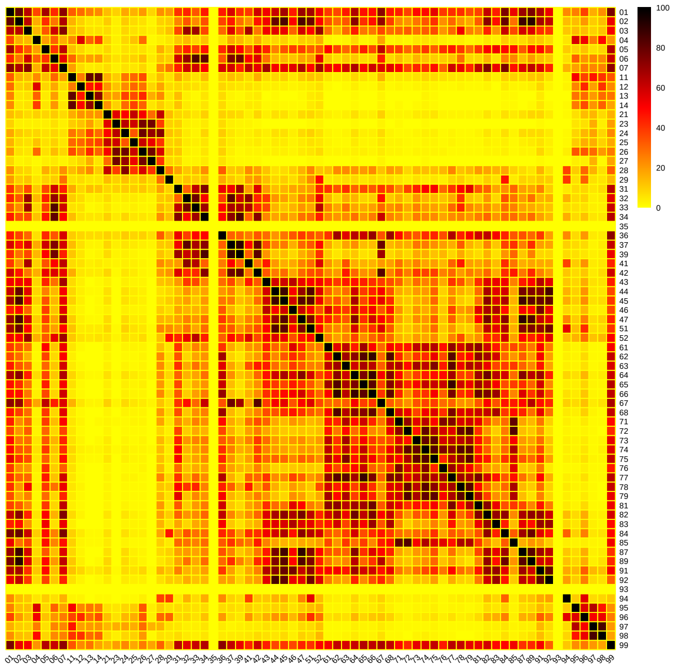

Figure S5: Knowledge relatedness over time. We focus on a subset including articles published from 2000 to 2009 (last 10 years in our data) to evaluate the evolution of the physics knowledge space over time. Despite a slightly general increase of interdisciplinarity, subject proximity indicates a stable structure among sub-fields.

a 1980 - 1989

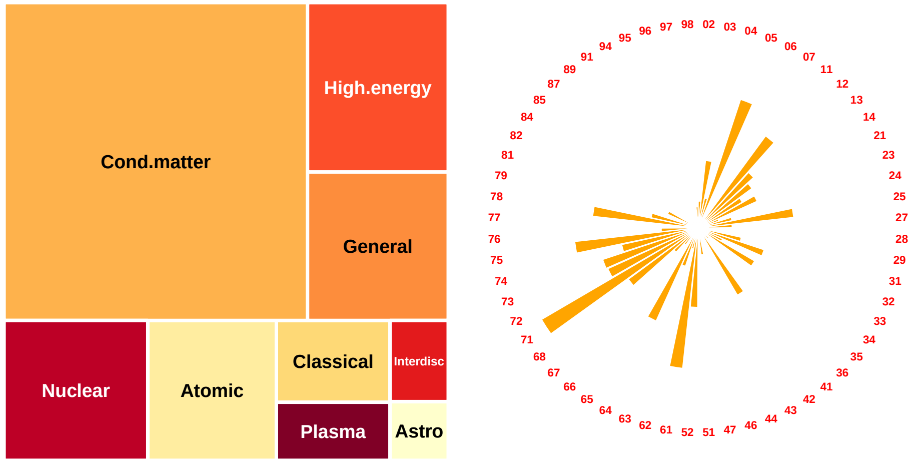

b 1990 - 1999

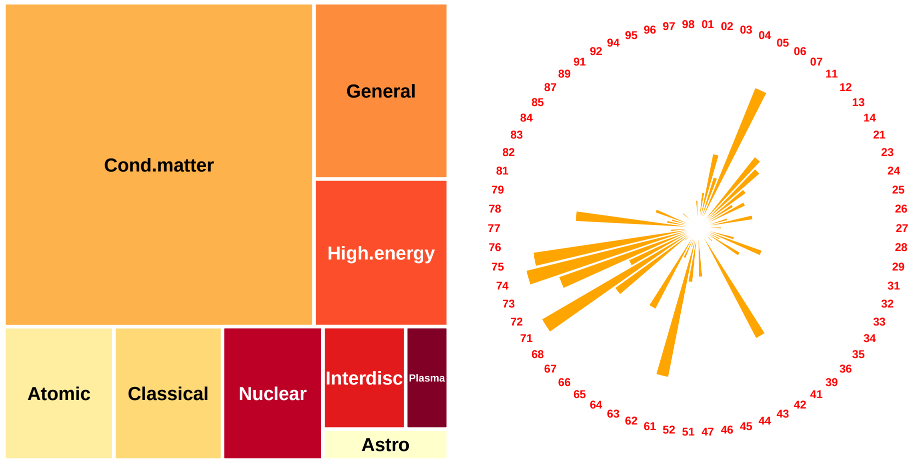

c 2000 - 2009

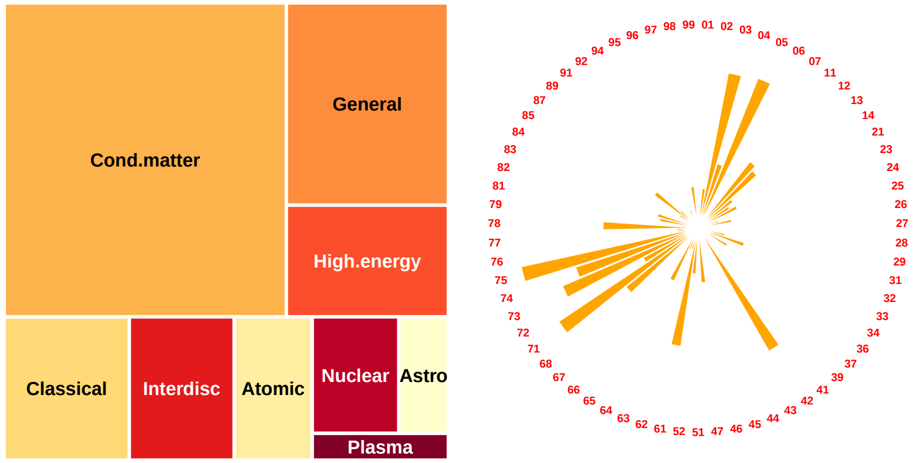

Figure S6: Popularity of fields and sub-fields through decades. The plots compare the popularity of physics fields and sub-fields over time (i.e., number of articles assigned to a given field/sub-field).

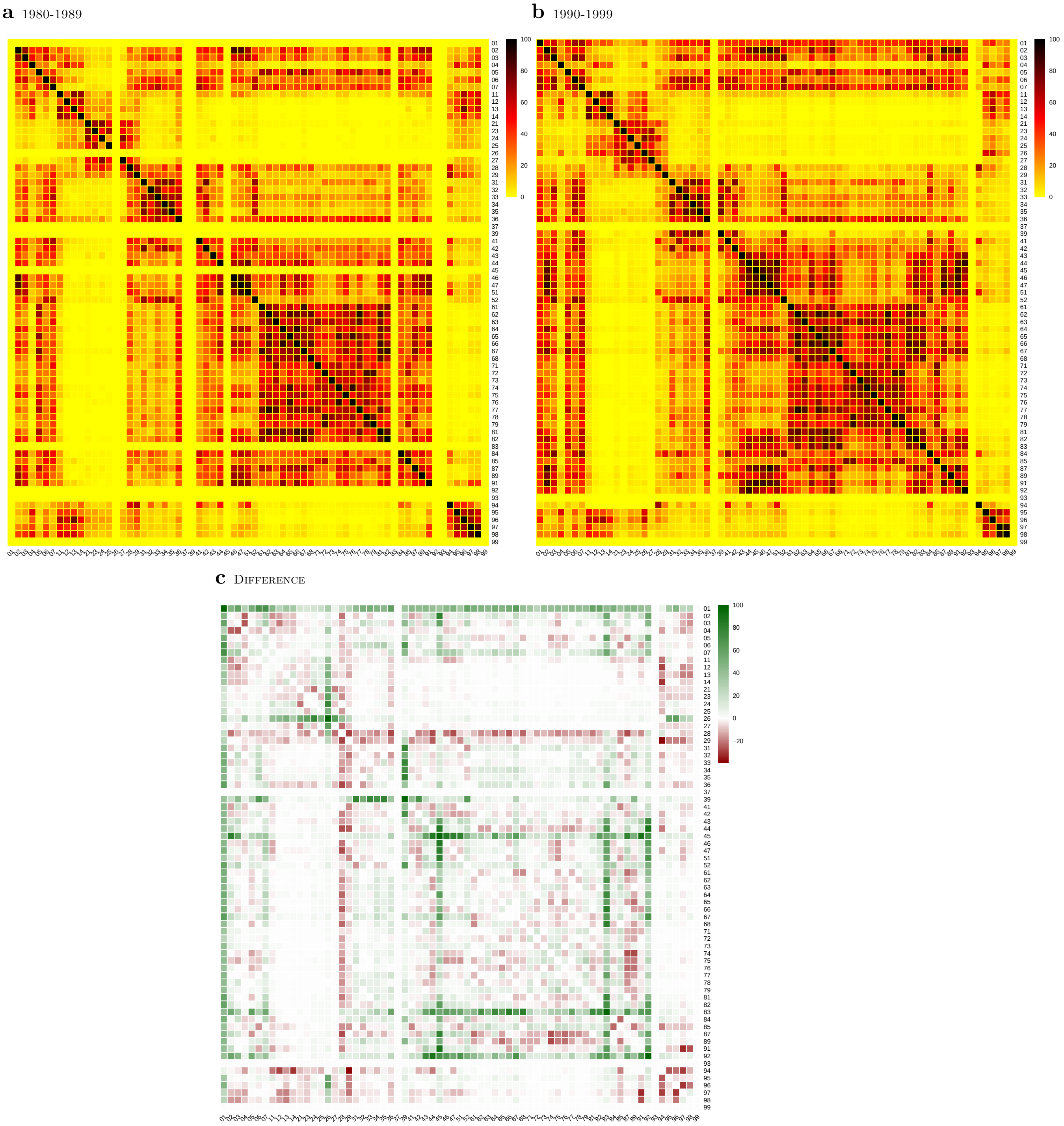

Figure S7: Knowledge relatedness evolution over the first two decades. The top panels show the cosine similarity matrix between two-digit PACS in two decades, while the bottom panel shows their difference.

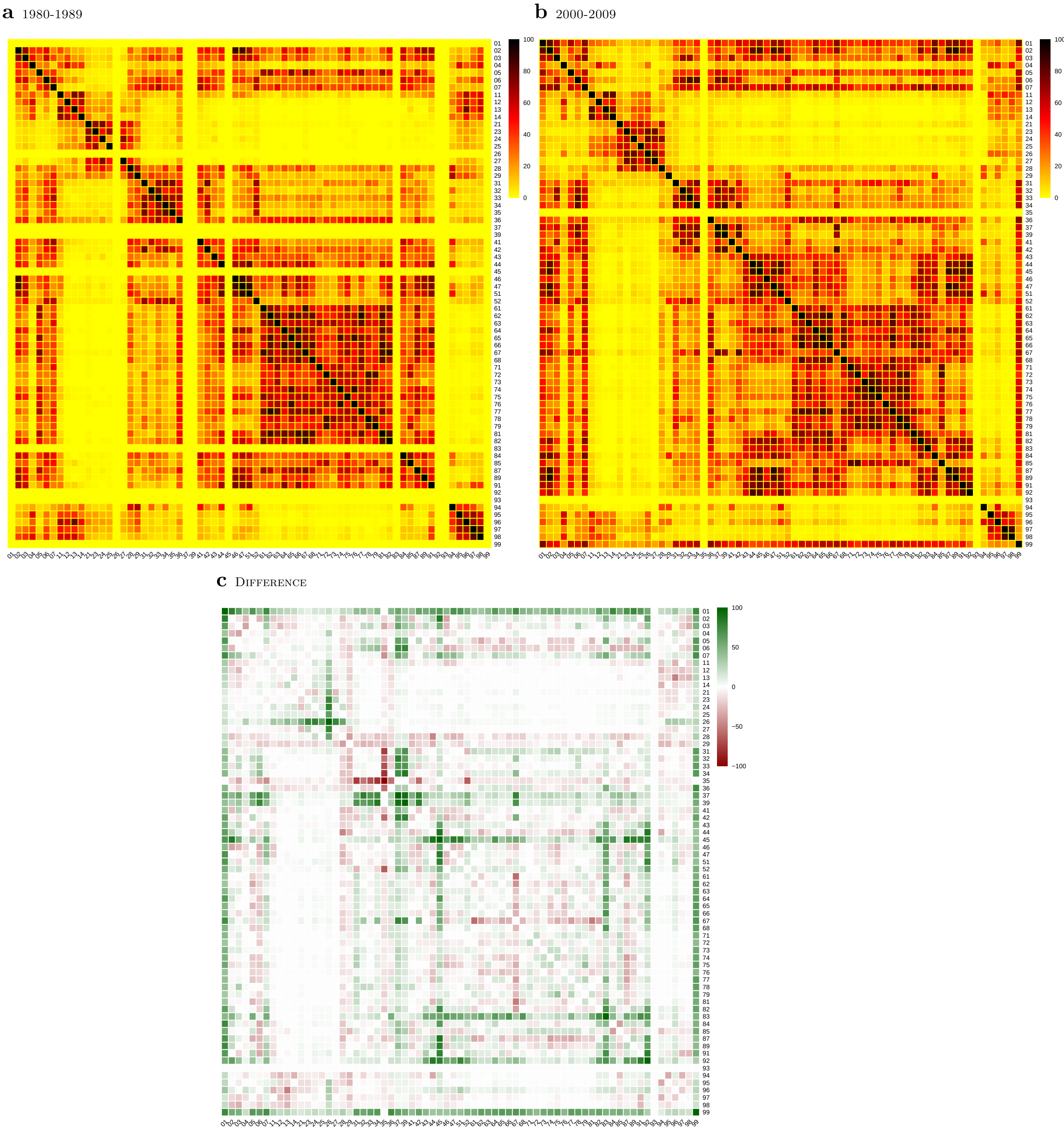

Figure S8: Knowledge relatedness evolution over three decades. The top panels show the cosine similarity matrix between two-digit PACS in two decades, while the bottom panel shows their difference.

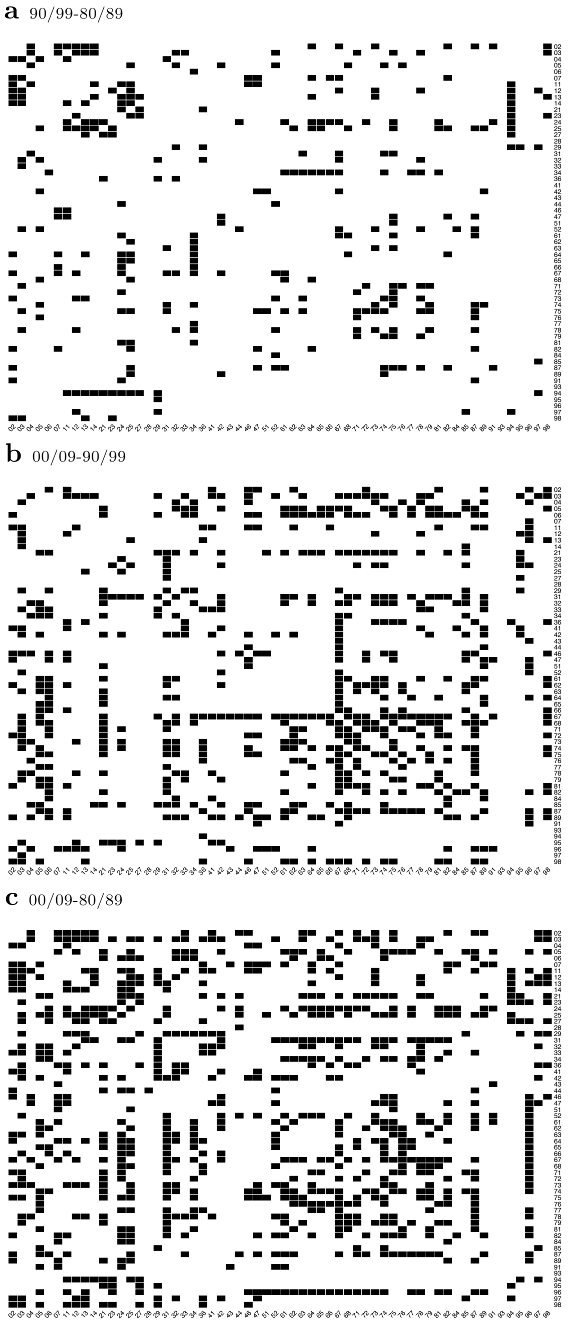

Figure S9: Bootstrap validation. Bootstrap validation of the difference matrices computed over decades by sampling with replacement and generating 1,000 additional difference matrices (showing only PACS codes present in each decade). The confidence interval ($\alpha = 0.05$) for each element of the matrix assesses the statistical significance (elements in black), taking into account multiple hypothesis testing correction (Bonferroni correction).

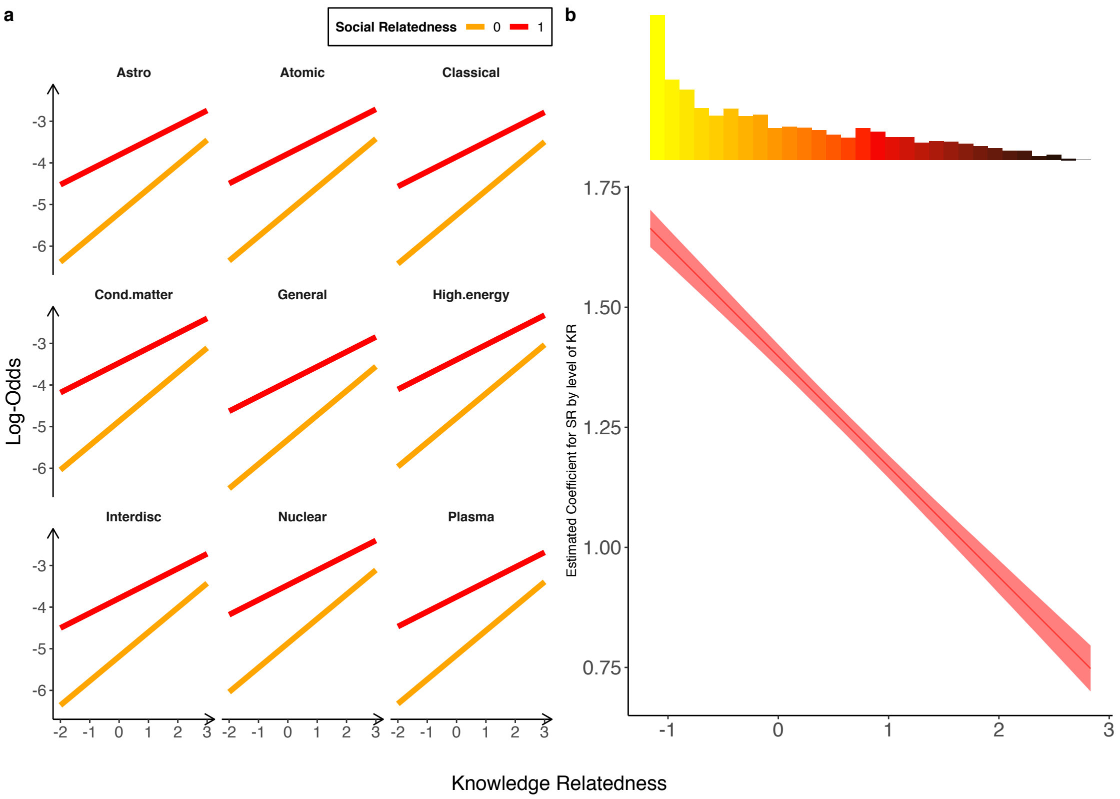

Figure S10: Scientists' research portfolio diversification - constrained diversification. (a) Log-odds as a function of (binary) social relatedness and (standardized) knowledge relatedness, accounting for multiple control variables. (i). (b) Estimated coefficient for social relatedness conditional on knowledge relatedness, and distribution of knowledge relatedness. The analysis is performed considering only truly unexplored sub-fields (see text).

a $Y_{t-1} = \alpha + \beta KR_{t-2} + \gamma SR_{t-2} \ + \zeta(KR_{t-2} \times SR_{t-2}) + \delta field\ core + \epsilon$

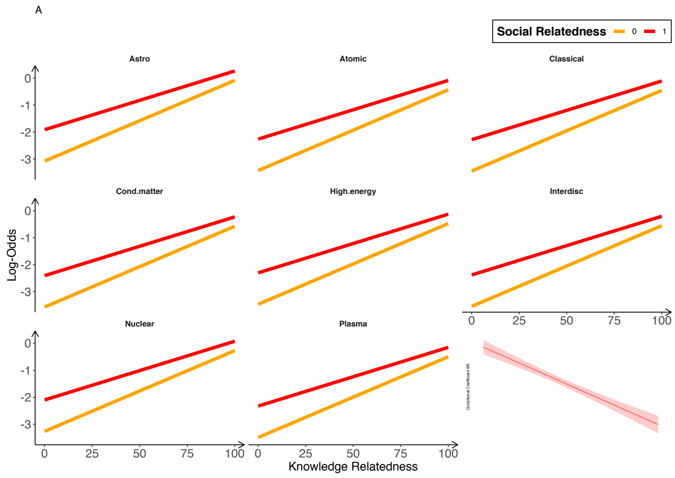

b $Y_{t} = \alpha + \beta KR_{t-1} + \gamma SR_{t-1} \ + \zeta(KR_{t-1} \times SR_{t-1}) + \delta field\ core + \epsilon$

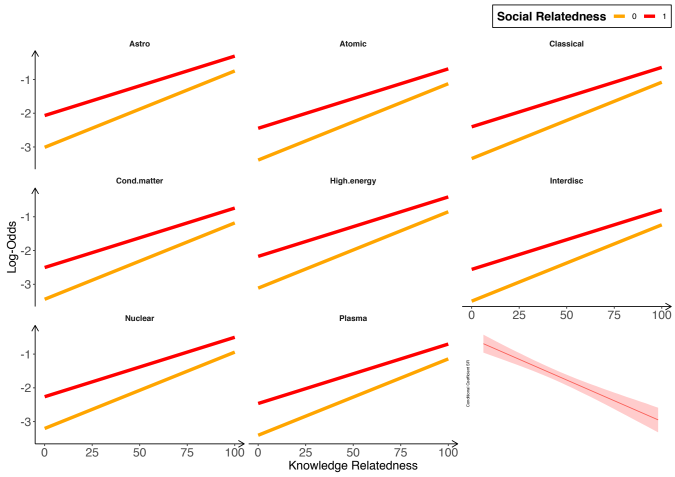

c $Y_{t} = \alpha + \beta KR_{t-2} + \gamma SR_{t-2} \ + \zeta(KR_{t-2} \times SR_{t-2}) + \delta field\ core + \epsilon$

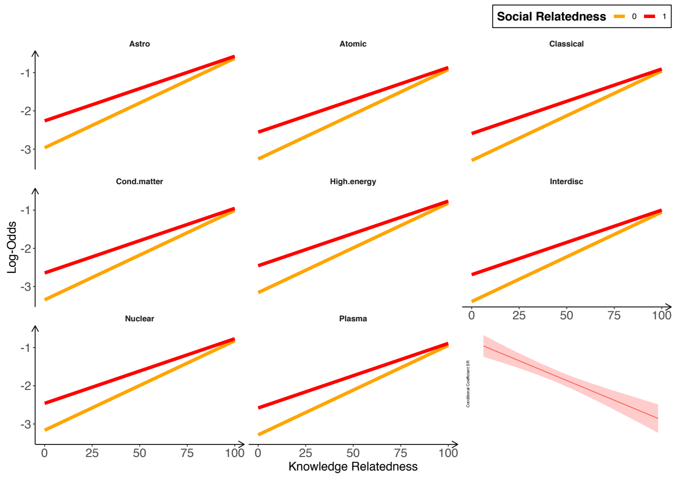

Figure S11: Models with lagged variables. Log-odds as function of social and (standardized) knowledge relatedness and (bottom right panel), estimated coefficient for social relatedness conditional on (standardized) knowledge relatedness.
